# Examining the links between vitamin C and schizophrenia spectrum disorders: a systematic review and meta-analysis on circulating levels and supplementation outcomes

**DOI:** 10.3389/fnut.2026.1965059

**Published:** 2026-10-14

**Authors:** Gaatha Suresh, Jeevitha Gada Chengaiyan, Fatemeh Dabaghzadeh, Faraz Ahmad

**Affiliations:** 1 School of Bio Sciences and Technology (SBST), Vellore Institute of Technology (VIT), Vellore, India; 2 Department of Clinical Pharmacy, Faculty of Pharmacy, Kerman University of Medical Sciences, Kerman, Iran

**Keywords:** antioxidant, ascorbate, psychosis, schizophrenic disorders, vitamin C deficiency

## Abstract

**Introduction:**

Schizophrenia (SCZ) is a debilitating psychiatric condition characterized by psychotic symptoms such as hallucinations, delusions, and disorganized thought. Recent evidence suggests its association with deficiency of various micronutrients. Vitamin C (vitC) is a key endogenous metabolite with potent antioxidant and neuroprotectant actions. However, the relationship between vitC deficiency and SCZ remains inconclusively established, both at the epidemiological and clinical levels, hindering its potential utility as a biomarker and a supplementary therapeutic. The purpose of this systematic review and meta-analysis was to investigate whether circulating levels of endogenous vitC are altered in SCZ subjects, and to examine the effect of exogenous vitC supplements on disease outcomes.

**Methods:**

Systematic searches for pertinent primary peer-reviewed studies on scholarly databases identified 19 studies. Newcastle-Ottawa scale and Methodological Index for Non-Randomized Studies were applied to judge the quality of studies of the case–control and supplementation trials, respectively. Random-effects restricted maximum likelihood meta-analysis models were employed for addressing the two specific aims of the study using standardized mean difference (SMD) as the point estimate.

**Results:**

SCZ patients showed significantly lowered vitC levels compared to the controls, with the pooled SMD being −1.046 [−1.459, −0.632]. Further, vitC supplementation was found to significantly reduce psychotic symptoms by −1.632 [−2.473, −0.791] units across a total of 6 datasets.

**Discussion:**

VitC deficiency appears to be a consistent feature of SCZ across diverse populations and its supplementation may be considered as a safe, inexpensive adjunct to antipsychotic therapy.

## 1 Introduction

Although low in prevalence, schizophrenia (SCZ) is a debilitating mental health condition with widespread emotional, social, and cognitive dysfunctions (1). The major symptomatic hallmarks of SCZ include hallucinations and delusions (positive symptoms) and emotional blunting, apathy and amotivation (negative symptoms). The psychotic phenotype along with social and cognitive dysfunctions result in considerably reduced functionality across almost all domains of day-to-day activities (2). Origins of SCZ are multifactorial and likely involve an intricate interplay of genetic, environmental, and life style factors (3). Recent evidence indicates significant contribution of diet to SCZ pathophysiology (4). Indeed, diet is a potentially modifiable factor that may help improve redox, inflammatory, synaptic, metabolic, and gut microbial dyshomeostasis. These disturbances are often found to be associated with SCZ patients (5). Nutritional supplementation, particularly with fatty acids and vitamins, has been investigated as an adjunctive strategy in SCZ (6, 7). However, the findings remain inconclusive, likely attributable to the limited number of studies, small sample sizes, heterogeneous dosing regimens, and variable intervention durations across trials. Nonetheless, vitamin deficiency appears to be a strong modifiable risk factor for SCZ development (8). Among the essential vitamins, ascorbic acid or vitamin C (vitC) is known to elicit significant roles as an endogenous antioxidant, anti-inflammatory, and neuroprotective agent (9). Unsurprisingly, significant mediation of vitC deficiency has been observed across a range of neurodevelopmental, psychiatric, and neurodegenerative conditions (10–12). Diminished circulating levels of vitamin C in SCZ cases have also been documented since the early psychiatric literature (13–15). This may be due to its reduced dietary intake (16) as well as its decreased assimilation (17). Interestingly, reduced intake of vitC is thought to be associated with increased odds of developing SCZ (18). Further, a Mendelian randomization-based causal relationship between SCZ and vitC supplementation has been propounded (19). Given these evidences, it is relevant to evaluate whether exogenous vitC supplementation can aid in improvement of disease outcomes, particularly by enhancing the therapeutic efficacy of antipsychotics. In this regard, previous case reports indicate that vitC may have therapeutic benefits for SCZ cases, likely in a case-specific manner (20, 21). Indeed, there have been propositions for vitC supplementation as a therapeutic adjuvant in the management of SCZ (22, 23); however, this has not always found support (24, 25).

Despite indications of links between vitC status and SCZ etio-pathophysiology, a comprehensive statistical synthesis across SCZ subjects with diverse demographic characteristics has been lacking. Previous attempts at such synthesis (26–28) have yielded largely inconclusive findings. The current systematic review and associated meta-analyses are aimed to address this research gap. Relying on primary data from multiple research groups across diverse demographical and clinical settings, we first aimed to evaluate the changes in the endogenous levels of vitC in SCZ cases, compared to controls. Additionally, we examined the effects of adjuvant vitC supplementation on disease symptomatology in SCZ cases.

## 2 Materials and methods

### 2.1 Research objectives and systematic literature search

The specific objectives of this meta-analysis were (i) to measure the differences in endogenous circulating levels of vitC between SCZ subjects and controls, and (ii) to assess the effect of vitC supplementation on the symptomatic severity in SCZ cases. Relevant primary research articles were retrieved from online scholarly web-databases (viz., PubMed and Google Scholar), to address the objectives detailed above in accordance with the Preferred Reporting Items for Systematic Reviews and Meta-Analyses (PRISMA) guidelines (Supplementary Table 1). The keywords used were {(“vitamin C” OR “ascorbate” OR “ascorbic acid” OR “hypovitaminosis C” OR “vitamin C deficiency” OR “vitamin C insufficiency” OR “vitamin C supplementation”) AND (“schizophrenia” OR “schizophrenia spectrum disorders” OR “schizophrenic disorders”)}. Only articles published between 1975 and April 2026 were included in the study. Studies citing the chosen papers and the references presented were also examined thoroughly to ensure that no relevant articles were overlooked during the web search.

The PICO scheme followed was: (i) Population (P): subjects with confirmed diagnosis of schizophrenia spectrum disorders (referred to as the SCZ group), (ii) Intervention (I): measurement of circulating (plasma/serum) endogenous levels of vitC OR oral supplementation of exogenous vitC either as a single or adjuvant therapeutic, (iii) Comparison (C): cases with no diagnosis of SCZ, including healthy controls, (iv) Outcome (O): difference in absolute vitC levels between SCZ cases and controls OR changes in disease outcome measures in SCZ cases before and after exogenous vitC supplementation. Based upon this scheme, the selection of primary studies adhered to specific exclusion and inclusion criteria to ensure accurate conclusions for the meta-analyses conducted.

The inclusion criteria were quantification of plasma/serum levels of vitC in SCZ cases and in the controls or measurement of psychotic symptoms in SCZ cases at baseline and after vitC supplementation. Studies that did not report specific data for SCZ cases, e.g., those with combined data for all psychotic cases, were excluded. Measurement assay for vitC levels, patient type (in- or out-patient), study design and ethnicity were not used to judge the inclusion or exclusion of the studies. However, research published in languages other than English was not included. This was largely due to the inability of the authors to fully comprehend and extract relevant data from non-English studies. While letters to editors and conference proceedings were considered to be eligible for selection, case reports, review articles, animal studies, and editorials were excluded. Initial retrieval of studies was individually performed by the authors, GS and FA. Final verification of titles, abstracts and full-texts of the final selected papers was done by authors, FA, FD and JGC in order to ensure their pertinence with respect to the defined goals.

### 2.2 Data extraction and study quality assessment

Extraction of data from the final set of primary studies was performed in a structured and homogeneous manner as per a pre-formulated scheme. Initial data extraction was performed by authors, GS and FA, and re-checked by JGC. The major categories of data extracted were first author and year, country of research, study design, sample sizes, specifications for cases (and controls), age and gender of cases/controls, SCZ diagnostic method, vitC measurement assay, and vitC levels in cases and controls (Supplementary Table 2). For supplementation studies, dose and duration of vitC intervention, instrument for scoring SCZ symptoms, and changes in psychotic scores in supplemented and non-supplemented groups were extracted (Supplementary Table 3).

The quality of the studies included in the meta-analysis was assessed using recognized scoring systems. The Newcastle-Ottawa scale (NOS) was used to evaluate case–control designs which evaluated the differences in vitC levels between SCZ cases and controls (29). The “non-response rate” item of the “exposure” domain, which evaluates potential selection bias arising from differential non-participation, was ignored for all included case–control studies. As these studies were hospital-based and cases and controls were directly enrolled from inpatient/outpatient clinical settings, the information on non-response rate was neither provided for cases nor for controls, making the NOS item inapplicable. To ensure methodological integrity for longitudinal studies on vitC supplementation, we implemented the Methodological Index for Non-Randomized Studies (MINORS) for non-comparative studies (30). As opposed to single-arm studies without a control/placebo group, for comparative studies, four items pertaining to the control group were additionally used to adjudge publication quality. Visual presentation of the risk of bias was based upon traffic-light and summary plots generated on an R-based web portal, risk-of-bias visualization (robvis) (31). Study quality assessment was performed by GS and FA, and confirmed by FD.

### 2.3 Meta-analyses

Pooled standardized mean difference (SMD) was chosen as the summary measure to effectively represent the changes in vitC levels in SCZ cases, compared to controls; as well as for the depiction of improvements in psychotic symptom scores in SCZ subjects supplemented with exogenous vitC. Restricted maximum likelihood (REML) method on a random-effects model was used for pooling data from the contributing studies. REML was chosen as simulation results indicated it as the least biased method of inter-study heterogeneity estimation when study numbers are small (32). A random-effects model was preferred over the fixed-effects model because of the demographical and methodological inter-study heterogeneity (33). Confidence intervals were presented at 95% confidence and statistical significance was tested using a two-tailed Z test with a significance threshold of 0.05. Inter-study heterogeneity was estimated by Cochran’s *Q* analysis, with the *τ^2^* statistic representing the variance in the summary measures. *I^2^* statistic was also estimated to determine the proportion of dispersion due to variance in true effects. Graphical presentation of inter-study heterogeneity was depicted as a Baujat plot. All statistical analyses and graphical creations were performed on metafor-R-based JASP software (34).

### 2.4 Sensitivity analysis

Case-wise sensitivity analyses were conducted to identify potential outlier studies, with standardized residual values, difference in fits (DFFITS), Cook’s distance, and the change in heterogeneity effected upon each study’s removal serving as diagnostic parameters. Lastly, robustness of the overall effect size was determined by applying the Rosenthal’s and Rosenberg’s fail-safe *N* estimation methods. The fail-safe *N* values depict the number of null studies required to reduce the pooled summary measure to non-significance, without and with accounting for study weights, respectively.

### 2.5 Publication biases

Statistical evaluation of publication bias due to small-study effects was carried out using Begg’s rank correlation test as well as Egger’s regression analysis. To further explore the possibility of missing studies, Duval and Tweedie’s trim-and-fill procedure was applied. This method identifies hypothetical studies missed due to publication bias and imputes them for recalculation of an adjusted pooled effect size. Graphical presentation of publication bias, along with any studies missed due to publication bias, was done using a funnel plot.

## 3 Results

### 3.1 Screening, data abstraction, and publication quality

Based upon the PICO scheme and applying the pre-set inclusion and exclusion criteria, a final set of 13 and 6 studies were identified for evaluating the differences in vitC levels between SCZ cases and controls, and for examining the changes in psychopathological outcomes in SCZ cases before and after vitC supplementation, respectively (Figure 1). Some of the studies that initially appeared eligible for inclusion for the final analysis were subsequently excluded. These included studies which did not report specific data for SCZ cases; notable examples were Myken (35), Shahini (36), and Bari (37). One study, Singh (38) compared vitC levels in SCZ cases under two different antipsychotic treatments (haloperidol vs. olanzapine); as no controls were included, the study was excluded. Further, Cha (16), Kim (18), Konarzewska (39), and Strassnig (40) compared dietary intake of vitC between SCZ cases and controls but did not report its circulating levels; therefore, these were excluded. One study, Issa (41) reported vitC levels in cerebrospinal fluid, as opposed to serum/plasma reported by others, and was therefore excluded from the analysis. Case reports (e.g., (20, 21)) and studies which did not provide explicit numerical information (e.g., (42)) were also excluded. Finally, one study, Surapaneni (43) which reported physiologically implausible levels of vitC was also excluded.

**Figure 1:**
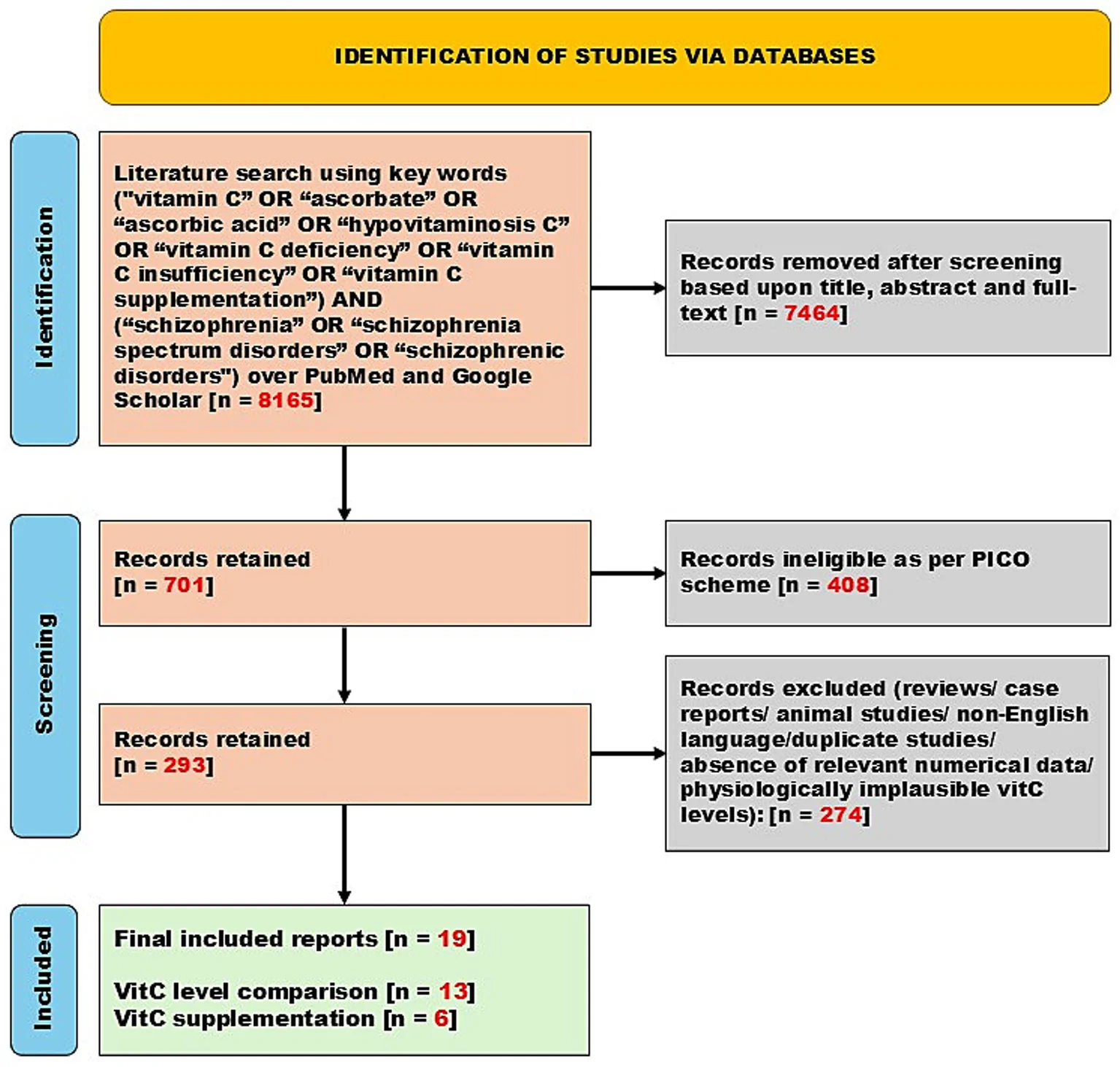
Flowchart outlining the approach to study selection and the sequential procedures employed in the systematic identification of relevant research.

Similarly, for vitC supplementation studies, records which did not report changes in disease outcomes were excluded. These included studies which evaluated assimilation of exogenously delivered vitC in SCZ cases by monitoring its levels in plasma and urine samples (17). Subotičanec (44) which was included for SMD analysis also reported vitC supplementation data; however, this was again focused on urinary excretion of the vitamin. Therefore, like previous studies from the same group, this was also not included in the final list of supplementation studies. Michael (45) and Pratiwi (46) only reported changes in tardive dyskinesia symptoms and immune parameters in SCZ subjects supplemented with vitC. Likewise, Ingole (47) and Zhang (48) focused on changes in metabolic parameters. All these studies were therefore not included in the final analysis. Vaughan (49) used Brief Symptom Inventory (BSI) for disease outcome measure, but did not provide actual scores; therefore, it was excluded. Straw (50) and Sivrioglu (51) measured the psychotic symptoms in SCZ cases with and without vitC supplementation, but graphically represented the changes, without providing actual numerical data. Because of the inability to reliably extract the data, we excluded these studies. Likewise, although Bentsen (52) used the widely employed Positive and Negative Syndrome Scale (PANSS) for evaluating the changes in the disease outcomes upon vitC supplementation, they did not report relevant values for the SMD calculation.

The quality of all primary studies, including 13 case–control studies and 6 longitudinal studies, was systematically evaluated using the NOS and MINORS schemes, respectively. For NOS, the non-response rate item was not reported in any of the included studies, reflecting the predominantly hospital-based, direct-enrolment design typical of these case–control studies, rather than a true methodological deficiency. Among the case–control studies, 10 studies were adjudged to be of low risk, while 3 were adjudged to have moderate risk (Figure 2). Regarding MINORS, two longitudinal studies (53, 54) did not include an untreated comparator group. The rest of the 4 studies were controlled trials. The overall risk of bias for these 6 longitudinal studies was either low (*n* = 3) or moderate (*n* = 3; Figure 3).

**Figure 2:**
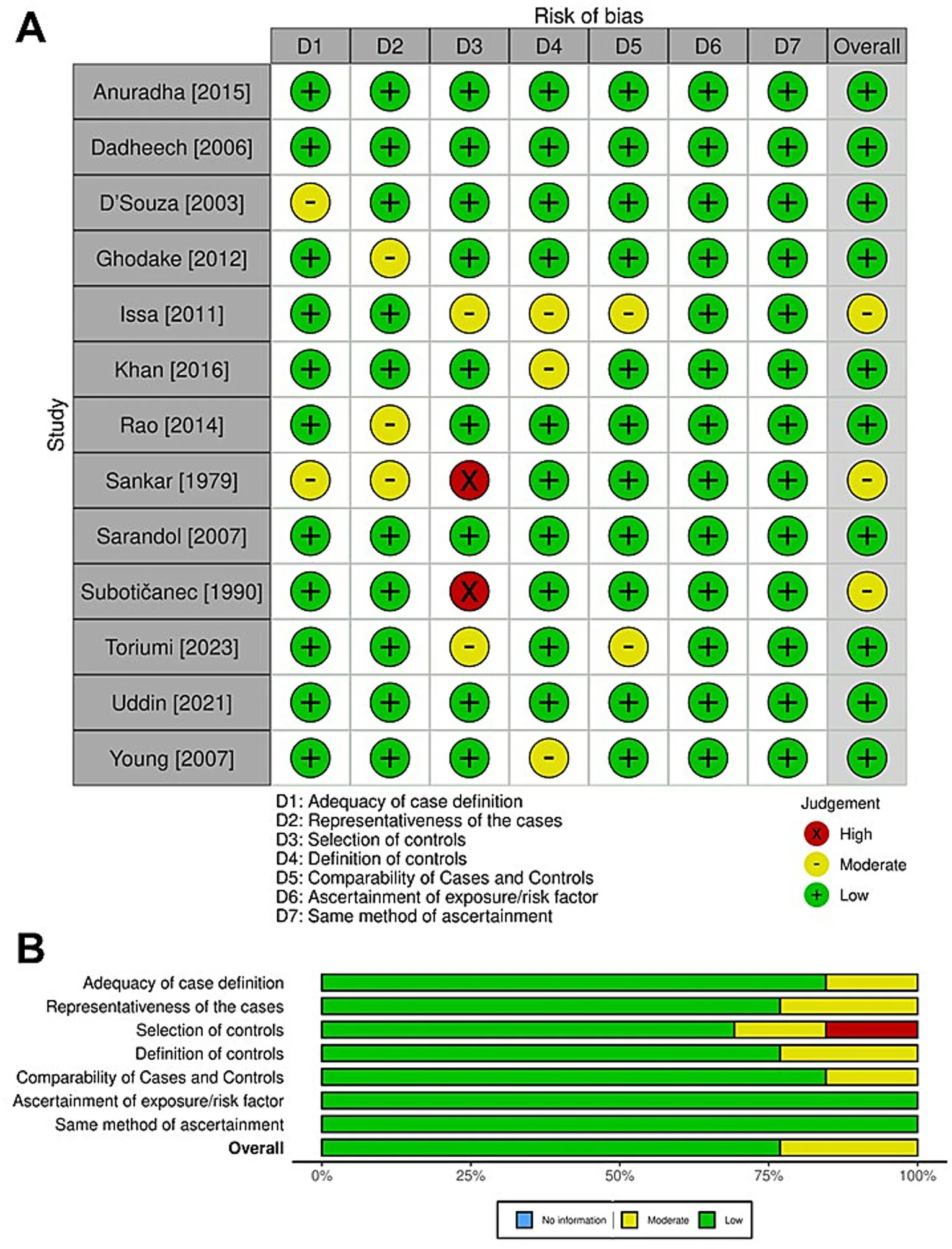
Traffic-light **(A)** and summary **(B)** plots illustrating the risk of bias for case-control studies included for the determination of the differences in vitC levels between SCZ cases and controls.

**Figure 3:**
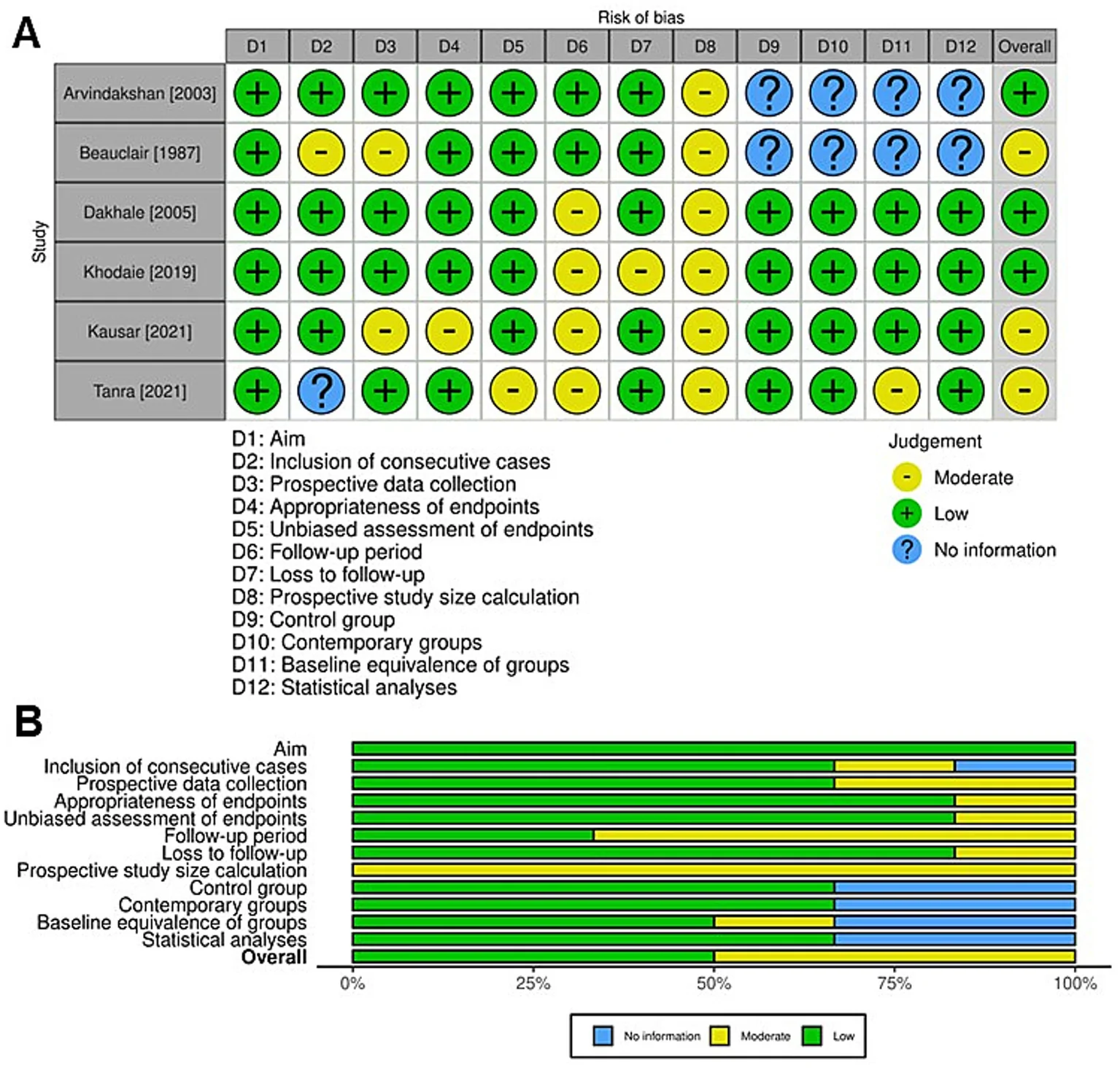
Traffic-light **(A)** and summary **(B)** plots for the risk of bias for longitudinal trials examining the effects of vitC supplementation on disease outcomes in SCZ cases.

### 3.2 SCZ cases show significant reductions in endogenous vitC levels

Thirteen case–control studies (Supplementary Table 4), enrolling a total of 884 SCZ subjects and 529 controls, were eligible for evaluating the differences in the circulating levels of vitC. The pooled SMD (SCZ minus controls) was estimated at −1.628 [95% CI, −2.481, −0.775], and this was statistically significant (*Z* = −3.74, *p* < 0.001). Significant heterogeneity amongst contributing studies was detected by Cochran’s *Q* analysis (*Q* = 305.51, dof = 12, *p* < 0.001), with the *τ^2^* and *I^2^* statistics estimated to be 2.286 and 97.25%, respectively. To identify the sources of this high heterogeneity, case-wise sensitivity diagnostic assessments were performed. This indicated Khan (75) and Rao (76) as potentially influential studies (Supplementary Figure 1; Supplementary Table 5). As these two studies appeared to contribute disproportionately to the reduced vitC levels observed in the SCZ cases, the meta-analysis was re-conducted following their exclusion.

The final set for the re-performed meta-analysis therefore included 11 studies comprising 731 SCZ subjects and 439 controls (Table 1). The direction of the pooled effect remained consistent after the exclusion of the two potential outlier studies, with the pooled SMD (SCZ minus controls) in vitC levels found to be −1.046 [95% CI: −1.459, −0.632]. Further, the statistical significance of the effect remained unaltered (*Z* = −4.95, *p* < 0.001; Figure 4A). However, significant heterogeneity amongst contributing studies was again detected by Cochran’s *Q* analysis (*Q* = 70.40, dof = 10, *p* < 0.001), with the *τ^2^* and *I^2^* statistics observed to be 0.309 and 83.86%, respectively. Both Egger’s regression (*t* = −2.094, *p* = 0.066) and Begg’s rank correlation (*τ* = −0.236, *p* = 0.359) analyses indicated no significant evidence of publication bias. However, Duval and Tweedie’s trim and fill method predicted that one study was missed due to publication bias (Figure 4B). Nevertheless, inclusion of this hypothetical study was predicted to change the adjusted pooled SMD only marginally to −0.932 [95% CI: −1.304, −0.559], without affecting statistical significance (*Z* = −4.90, *p* < 0.001). Rosenthal’s fail-safe *N* estimate of 503 indicated the requirement of 503 null studies to render the overall effect non-significant, further confirming the robustness of the results. Rosenberg’s approach yielded a similarly large fail-safe *N* of 285, again supporting the stability of the results. Lastly, case-wise sensitivity diagnostics did not identify any studies with potentially disproportionate influences on the summary measure (Figure 4C, Table 2).

**Table 1:** Consolidated list of studies evaluating differences in vitC levels between SCZ cases and controls, following the removal of outlier studies.

S. No.	First author (Year, Reference)	Country of study	Sample size		VitC levels (mg/dL; mean ± SD)	
			Ctrl	SCZ	Ctrl	SCZ
1	Anuradha (2015) (64)	India	30	90	1.19 ± 0.14	1.04 ± 0.14
2	Dadheech (2006) (65)	India	40	58	1.26 ± 0.43	0.75 ± 0.21
3	D’Souza (2003) (66)	India	18	14	0.8078 ± 0.51965	0.2904 ± 0.196
4	Ghodake (2012) (67)	India	30	30	0.90 ± 0.21	0.63 ± 0.14
5	Issa (2011) (68)	Iraq	30	70	3.41 ± 0.83	1.75 ± 1.01
6	Sankar (1979) (69)	United States	24	97	1.045 ± 0.312	0.9208 ± 0.3948
7	Sarandol (2007) (70)	Turkey	35	40	0.983 ± 0.460	0.910 ± 0.627
8	Subotičanec (1990) (44)	Former Yugoslavia (Croatia)	35	35	0.6871 ± 0.5013	0.3886 ± 0.321
9	Toriumi (2023) (71)	Japan	117	218	1.4092 ± 1.4195	1.0603 ± 1.5959
10	Uddin (2021) (72)	Bangladesh	63	63	0.195 ± 0.043	0.133 ± 0.060
11	Young (2007) (73)	United Kingdom	17	16	0.961 ± 0.093	0.805 ± 0.069

Ctrl, controls; SCZ, schizophrenia; SD, standard deviation; vitC, vitamin C.

**Figure 4:**
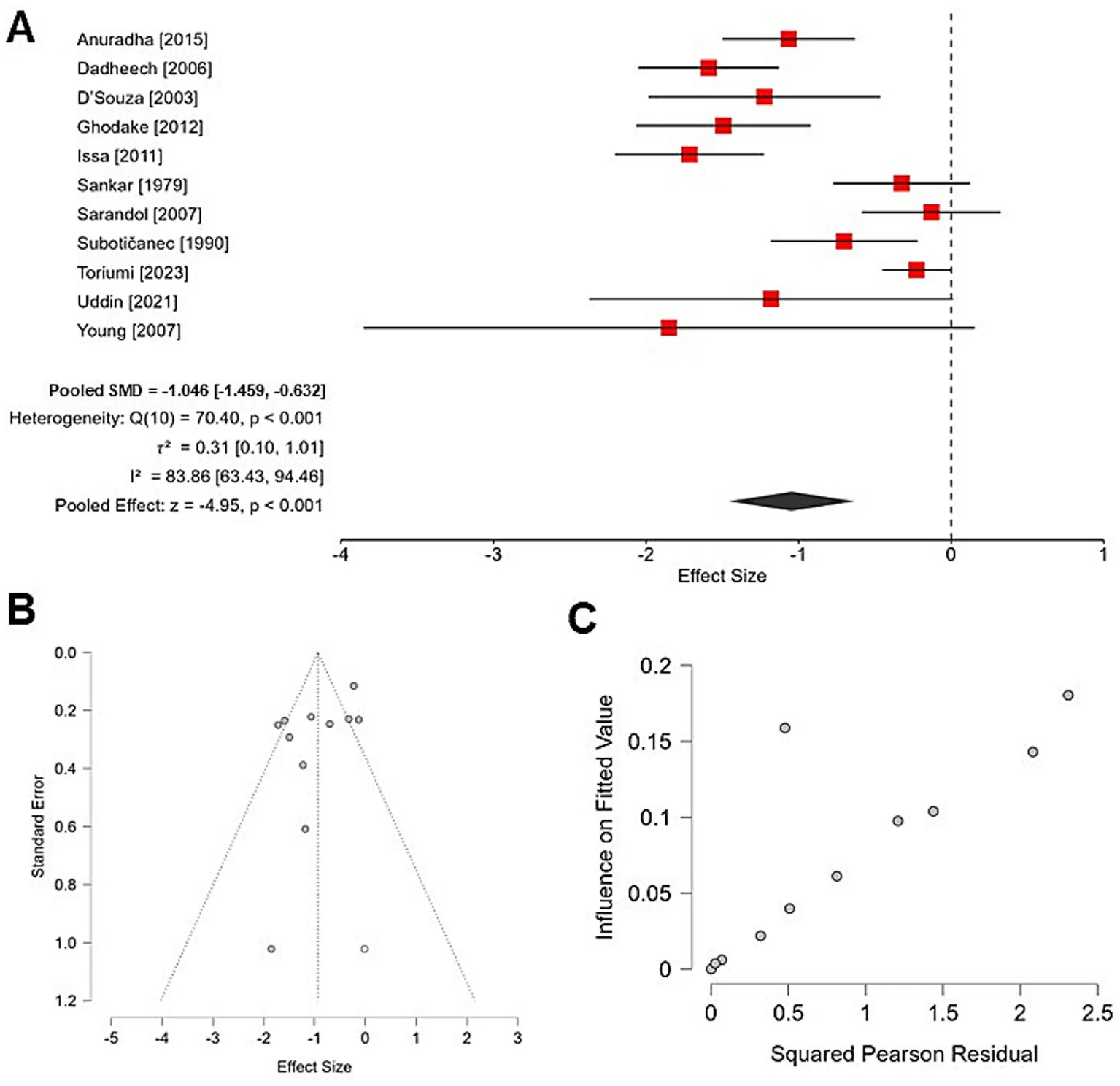
Meta-analytical assessment of the differences in the circulating vitC levels in SCZ cases versus controls, following exclusion of Khan (2016) and Rao (2014) as influential outliers identified in preliminary analysis (Supplementary Figure 1). **(A)** Forest plot shows an overall significant reduction in vitC levels in SCZ cases. **(B)** Funnel plot of the included studies (grey circles); a single study was predicted (open circle) to be missed due to publication bias in trim and fill analysis. **(C)** Baujat plot showing each study’s contribution to the overall heterogeneity against its influence on the pooled effect estimate.

**Table 2:** Case-wise diagnostics table for studies evaluating vitC level in SCZ subjects versus controls.

First author (Year, Reference)	Standardized residual	DFFITS	Cook’s distance	Covariance ratio	Leave-one-out		Influential
					*𝜏*	*Qe*	
Anuradha (2015) (64)	−0.031	−0.009	8.176 × 10^−5^	1.224	0.593	67.52	No
Dadheech (2006) (65)	−0.954	−0.263	0.066	1.086	0.539	54.91	No
D’Souza (2003) (66)	−0.268	−0.082	0.007	1.168	0.580	68.57	No
Ghodake (2012) (67)	−0.737	−0.212	0.045	1.127	0.558	62.72	No
Issa (2011) (68)	−1.203	−0.332	0.101	1.032	0.516	52.71	No
Sankar (1979) (69)	1.241	0.343	0.117	1.121	0.553	67.33	No
Sarandol (2007) (70)	1.654	0.452	0.188	1.042	0.520	63.59	No
Subotičanec (1990) (44)	0.557	0.157	0.027	1.211	0.589	70.40	No
Toriumi (2023) (71)	1.567	0.404	0.150	1.051	0.520	42.82	No
Uddin (2021) (72)	−0.172	−0.063	0.004	1.105	0.575	69.79	No
Young (2007) (73)	−0.749	−0.380	0.145	0.914	0.559	69.15	No

DFFITS, difference in fits.

It should be noted here that, in comparison to the initial analysis (*n* = 13 studies), the meta-analysis performed after removal of the two outlier studies retained both the direction and statistical significance of the pooled effect size indicator. While *I^2^* value was decreased from 97.25 to 83.86%, considerable heterogeneity still remained unexplained. Inter-study differences in a plethora of moderators, including disease duration, age of onset, treatment status, in-/out-patient status, age of cases/controls, and vitC assay type used, could potentially have contributed to the heterogeneity in effect sizes. However, subgroup analyses or meta-regression could not be performed due to severe limitations in the secondary data available.

### 3.3 Exogenous supplementation of vitC in SCZ cases is likely associated with improved disease outcomes

For the evaluation of the psychopathological improvements amongst SCZ cases upon vitC supplementation, six studies with seven datasets (Supplementary Table 6), recruiting a total of 145 SCZ cases, were identified. Of these, two (Arvindakshan (53) and Beauclair (54)) were single-arm studies with no comparative group. The rest of the four studies included respective control groups, amounting to 104 SCZ cases without vitC supplementation. Among these, one study, Kausar (55), did not state the pre-post values for the control group. Further, barring Beauclair (54) and Dakhale (56), which used Brief Psychiatric Rating Scale (BPRS), the other four studies employed PANSS for scoring psychotic symptoms, with Arvindakshan (53) additionally using BPRS. Tanra (57) reported changes in only the total negative symptom score (NSS) domain of the PANSS. We initially planned to perform two sets of meta-analytical assessments evaluating (i) within-group change in vitC-supplemented SCZ patients (after versus before supplementation) only, and (ii) between-group difference in this change when compared with control/placebo-treated SCZ patients (i.e., supplemented versus placebo SCZ groups, each assessed after versus before). While the former could be conveniently performed because of relative sufficiency of available data, the latter was abandoned due to the lack of studies (*n* = 3 datapoints; see Supplementary Table 3).

Within-group differences (post-supplementation minus baseline) in psychopathological scores were found to be significant, as indicated by the pooled SMD of −2.094 [95% CI: −3.235, −0.953] (*Z* = −3.60, *p* < 0.001; Supplementary Figure 2). Cochran’s assessment identified significant inter-study heterogeneity (*Q* = 85.04, dof = 6, *p* < 0.001); and the *τ^2^* and *I^2^* statistics were calculated to be 2.219 and 94.62%, respectively. To address the substantially large inter-study heterogeneity, case-wise sensitivity analysis was performed. This identified Tanra (57) as an influential study with disproportionately large contribution to the pooled point estimate (Supplementary Table 7).

To consolidate our results, the meta-analysis was re-conducted after excluding the outlier study. Five studies with six datasets, with a total of 118 SCZ cases under vitC supplementation, were used for the assessment (Table 3). The pooled SMD (post minus pre) in psychotic symptom scores was found to be −1.632 [95% CI: −2.473, −0.791] (*Z* = −3.80, *p* < 0.001; Figure 5A). The direction and statistical significance of the pooled effect remained consistent after exclusion of the outlier study (Tanra (57)). However, heterogeneity remained substantial even after its removal, as indicated by Cochran’s test (*Q* = 45.41, dof = 5, *p* < 0.001), and the *τ^2^* and *I^2^* statistics values of 0.977 and 89.54%, respectively. Both Egger’s (*t* = −1.979, *p* = 0.119) and Begg’s (*τ* = −0.733, *p* = 0.056) tests failed to identify publication bias. Correspondingly, trim and fill analysis also did not predict any potential study which could have been missed due to publication bias (Figure 5B). Rosenthal’s and Rosenberg’s fail-safe *N* values were estimated at 263 and 160, indicating general robustness of the pooled summary measure. Lastly, sensitivity analysis represented as case-wise sensitivity diagnostic measures did not identify any study with disproportionately large contribution to the pooled point estimate (Figure 5C, Table 4).

**Table 3:** Consolidated list of trials examining the effects of vitC supplementation in SCZ cases after removal of the outlier study.

S. No.	First author (Year, Reference)	Country	Study type	Sample size	VitC dose and duration	Disease outcome scoring instrument	Baseline total psychotic symptom score (mean ± SD)	Final total psychotic symptom score (mean ± SD)
1	Arvindakshan (2003) (53)	India	Single arm pre-post-trial	28	2 × 500 mg/day for 4 months	PANSS (TS)	80 ± 26.9	61 ± 22.7
						BPRS	45 ± 16.5	35 ± 13.7
2	Beauclair (1987) (54)	USA	Single arm pre-post-trial	13	Escalating doses for a period of 8 weeks: 1 g/day (weeks 1–2), 2 g/day (weeks 3–4), 4 g/day (weeks 5–6), 8 g/day (weeks 7–8)	BPRS	42 ± 4.5	34 ± 5.5
3	Dakhale (2005) (56)	India	Randomized, double-blind, placebo-controlled, pre-post-trial	20	500 mg/day for 8 weeks	BPRS	36.65 ± 5.78	19.30 ± 5.46
4	Khodaie (2019) (74)	Iran	Randomized, double-blind, placebo-controlled, pre-post-trial	22	2 × 500 mg/day for 4 months	PANSS (TS)	86.3 ± 18.9	65.1 ± 18.7
5	Kausar (2021) (55)	Pakistan	Randomized, placebo-controlled, pre-post-trial	35	500 mg/day for 8 weeks	PANSS (TS)	88.72 ± 18.39	46.93 ± 8.22

BPRS, Brief Psychiatric Rating Scale; PANSS, Positive and Negative Syndrome Scale; TS, total score; SD, standard deviation; vitC, vitamin C.

**Figure 5:**
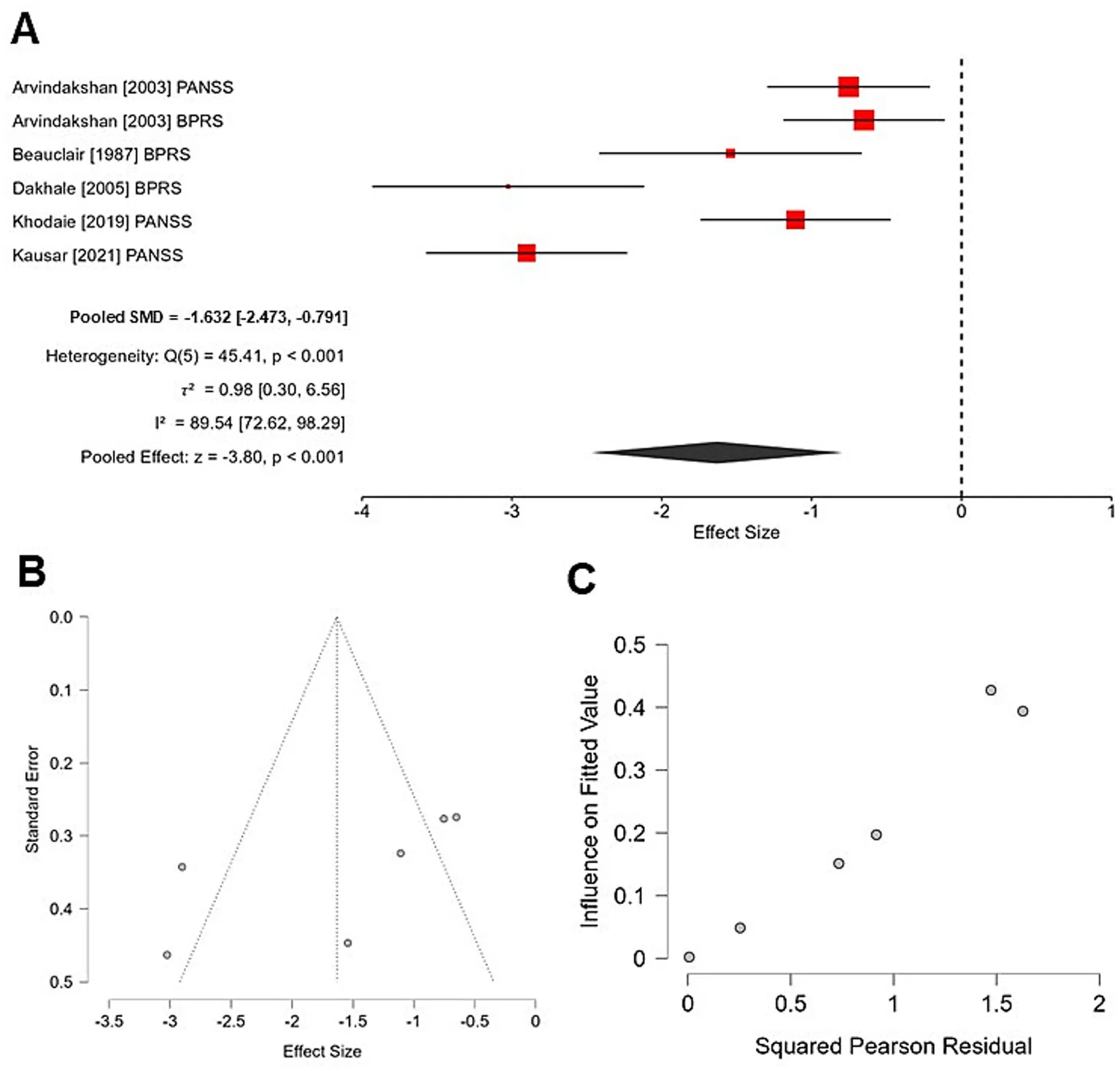
Meta-analytical assessment of the changes in SCZ psychopathology upon exogenous vitC supplementation. **(A)** Forest plot showing variable but consistent improvements in psychotic scores after vitC supplementation in SCZ cases, compared to baseline scores. **(B)** Funnel plot of the included datasets (grey circles); no potential studies were identified to be missed due to small-study effects by the trim and fill analysis. **(C)** Baujat plot depicting each dataset’s contribution to the overall heterogeneity, plotted against its influence on the pooled effect estimate.

**Table 4:** Case-wise diagnostics table for vitC supplementation trials.

First author (Year, Reference)	Standardized residual	DFFITS	Cook’s distance	Covariance ratio	Leave-one-out		Influential
					*𝜏*	*Qe*	
Arvindakshan (2003) (53) PANSS	0.925	0.428	0.191	1.260	1.011	38.24	No
Arvindakshan (2003) (53) BPRS	1.063	0.489	0.235	1.192	0.979	35.56	No
Beauclair (1987) (54) BPRS	0.087	0.051	0.003	1.472	1.114	45.29	No
Dakhale (2005) (56) BPRS	−1.559	−0.674	0.363	0.922	0.859	31.89	No
Khodaie (2019) (74) PANSS	0.513	0.242	0.069	1.426	1.086	44.43	No
Kausar (2021) (55) PANSS	−1.536	−0.718	0.393	0.919	0.847	22.51	No

BPRS, Brief Psychiatric Rating Scale; DFFITS, difference in fits; PANSS, Positive and Negative Syndrome Scale.

## 4 Discussion

The present systematic review and meta-analysis is unique in the context of SCZ research as it not only examines the alterations in the circulating plasma/serum vitC concentrations in individuals diagnosed with the condition, but also evaluates the impact of exogenous supplementation of this antioxidant vitamin on disease symptomatology. Given that oxidative stress and damage is a primary mechanism in the pathophysiology of SCZ (58, 59), it is relevant to assess the clinico-epidemiological links between SCZ and vitC, a major endogenous cellular antioxidant in the brain (10, 60). Previously published assessments report inconsistent findings regarding both circulating vitC levels (28) and the therapeutic benefits of vitC supplementation (25, 27) in SCZ patients. Hence, in this study, we aimed at an updated assessment of the links between vitC and SCZ pathophysiology, using specific data for the cases diagnosed with the disorder.

Our results show that SCZ patients exhibited significantly diminished endogenous vitC levels compared to controls across all studies included in the final set (*n* = 11 studies). This pattern was observed in diverse samples with different geocultural backgrounds indicating that hypovitaminosis C is likely a consistent feature of the condition. However, there was substantial inter-study heterogeneity, presumably due to variations in a wide range of factors, including diagnostic criteria, patient characteristics, disease stage, medication status, and vitC measurement methods. Unfortunately, due to limitations of the secondary data reported in the original studies, subgroup or meta-regression analyses could not be performed in a reliable manner. Subsequent analysis detected absence of significant publication bias in Egger’s and Begg’s tests. On the other hand, trim and fill analysis uncovered a hypothetical study missed due to publication bias. The summary measure estimated upon inclusion of this missed study was predicted to marginally change from −1.046 [95% CI: −1.459, −0.632] to an adjusted SMD of −0.932 [95% CI: −1.304, −0.559]. Moreover, the statistical significance was retained, as evident from the minor change in *Z* value from −4.95 (*p* < 0.001) to −4.90 (*p* < 0.001).

The reduced levels of vitC in SCZ cases should not be regarded merely as simple dietary deficiency or biochemical anomaly; rather, they are indicative of redox imbalance characterized by diminished endogenous antioxidant status and potentially a heightened state of oxidative stress and damage (61, 62). Nonetheless, it should be noted that our results do not implicate vitC deficiency as a causal factor in SCZ etiopathology. As is inherent to observational study designs, causal relationships cannot be established. Indeed, the relationship between vitC status and SCZ can be bidirectional as SCZ subjects often elicit dietary and metabolic dysfunctions (5, 63), which may result in vitC deficiency.

With regards to the analysis of data from vitC supplementation trials, our results indicate that exogenous provision of the vitamin may alleviate psychopathology in SCZ cases. Across five studies comprising six datasets, the psychotic symptom scores in SCZ cases were found to be significantly lowered after vitC intervention, with the pooled SMD estimated at −1.632 [95% CI: −2.473, −0.791]. However, heterogeneity was substantial, likely attributable to variations in the type of concurrent therapeutic regimens, dose and duration of vitC intervention, and underlying clinical and demographical statuses of the subjects. While there was no evidence of small-study effects or missing studies suggestive of publication bias, the high inter-study heterogeneity and the limited number of small trials mean that these findings should be interpreted solely as hypothesis-generating, rather than confirmatory. The pooled estimate is best regarded as indicative of a possible direction of effect, rather than definitive evidence of clinical benefit. While other researchers have propounded employment of vitC as a simple, safe, and inexpensive dietary component to complement standard therapy in SCZ patients (23), in our view, further adequately powered investigations are warranted to affirm the utilities of vitC as a potential adjunctive therapy for the management of SCZ.

Despite the promising findings of a significant association between vitC status and SCZ etio-pathophysiology, there are a few limitations which must be discussed. First, there was significant methodological, demographical, and clinical heterogeneity amongst eligible studies, likely contributing to the high *τ^2^* and *I^2^* values and limiting generalizability of the findings. Furthermore, the lack of relevant moderator information in most of the primary studies precluded meta-regression and subgroup analyses. Therefore, in view of the relatively low number of eligible studies and high inter-study heterogeneity, our results should be interpreted with caution. Most supplementation trials enrolled small samples, potentially limiting statistical power. As discussed previously (Methods section 2.2), the case-control studies were hospital based and did not provide information on the non-response rate, rendering the said item of the exposure domain of the NOS inapplicable. This may be considered an additional limitation. Moreover, due to substantial deficiency of primary data, we were unable to include comparative (un-supplemented control or placebo) groups in the analyses. Another limitation arising due to limited data was that we were unable to assess the impact of vitC intervention on specific subdomains of positive, negative, and general psychopathological symptoms. Lastly, most of the supplementation trials were short-term, typically lasting only for 8 weeks; hence the long-term effects remained obscure. Therefore, in order to solidify the initial positive finding observed here, we recommend the conduction of large-scale controlled and longitudinally extended trials in diverse populations for comprehensive evaluations of the efficacy of vitC supplementation on relapse rates as well as other clinical parameters such as cognitive function and quality of life. In addition, more nuanced research needs to be conducted in order to personalize therapy for patients, especially those with severe deficiency.

## 5 Conclusion

Clinico-epidemiological evidence supports vitC deficiency as a likely characteristic in SCZ pathophysiology. While the results from intervention trials are supportive of a potential benefit, they do not yet provide sufficient justification for recommending vitC supplementation as a standard adjuvant within treatment regimens. Future large-scale, well-designed controlled trials, employing standardized methodologies and analytic paradigms are needed to further clarify the relationship between vitC levels and SCZ etio-pathophysiology.

## Data Availability

The original contributions presented in the study are included in the article/Supplementary material, further inquiries can be directed to the corresponding author.

## References

[1] Charlson FJ Ferrari AJ Santomauro DF Diminic S Stockings E Scott JG . Global epidemiology and burden of schizophrenia: findings from the global burden of disease study 2016. Schizophr Bull. (2018) 44:1195–203. doi: 10.1093/schbul/sby058, 29762765 PMC6192504

[2] Mueser KT McGurk SR. Schizophrenia. Lancet. (2004) 363:2063–72. doi: 10.1016/S0140-6736(04)16458-1, 15207959

[3] Owen MJ Sawa A Mortensen PB. Schizophrenia. Lancet. (2016) 388:86–97. doi: 10.1016/S0140-6736(15)01121-6, 26777917 PMC4940219

[4] van Zonneveld SM Haarman BCM van den Oever EJ Nuninga JO Sommer IEC. Unhealthy diet in schizophrenia spectrum disorders. Curr Opin Psychiatry. (2022) 35:177–85. doi: 10.1097/YCO.0000000000000791, 35585755

[5] Rarinca V Vasile A Visternicu M Burlui V Halitchi G Ciobica A . Relevance of diet in schizophrenia: a review focusing on prenatal nutritional deficiency, obesity, oxidative stress and inflammation. Front Nutr. (2024) 11:1497569. doi: 10.3389/fnut.2024.1497569, 39734678 PMC11673491

[6] Chia SC Henry J Mok YM Honer WG Sim K. Fatty acid and vitamin interventions in adults with schizophrenia: a systematic review of the current evidence. J Neural Transm. (2015) 122:1721–32. doi: 10.1007/s00702-015-1451-z, 26354100

[7] Mitra S Natarajan R Ziedonis D Fan X. Antioxidant and anti-inflammatory nutrient status, supplementation, and mechanisms in patients with schizophrenia. Prog Neuropsychopharmacol Biol Psychiatry. (2017) 78:1–11. doi: 10.1016/j.pnpbp.2017.05.005, 28499901

[8] Bansal V Chatterjee I. Association of vitamins and neurotransmitters: understanding the effect on schizophrenia. Neurochem J. (2022) 16:39–45. doi: 10.1134/S1819712422010032

[9] Alberts A Moldoveanu E-T Niculescu A-G Grumezescu AM. Vitamin C: a comprehensive review of its role in health, disease prevention, and therapeutic potential. Molecules. (2025) 30:748. doi: 10.3390/molecules30030748, 39942850 PMC11820684

[10] Figueroa-Méndez R Rivas-Arancibia S. Vitamin C in health and disease: its role in the metabolism of cells and redox state in the brain. Front Physiol. (2015) 6:397. doi: 10.3389/fphys.2015.00397, 26779027 PMC4688356

[11] Moretti M Fraga DB Rodrigues ALS. Ascorbic acid to manage psychiatric disorders. CNS Drugs. (2017) 31:571–83. doi: 10.1007/s40263-017-0446-8, 28600627

[12] Kocot J Luchowska-Kocot D Kiełczykowska M Musik I Kurzepa J. Does vitamin C influence neurodegenerative diseases and psychiatric disorders? Nutrients. (2017) 9:659. doi: 10.3390/nu9070659, 28654017 PMC5537779

[13] Pitt B Pollitt N. Ascorbic acid and chronic schizophrenia. Br J Psychiatry. (1971) 118:227–8. doi: 10.1192/bjp.118.543.227 5576286

[14] Milner G. Ascorbic acid in chronic psychiatric patients —a controlled trial. Br J Psychiatry. (1963) 109:294–9. doi: 10.1192/bjp.109.459.294

[15] Subotičanec K. Vitamin C status in schizophrenia. Bibl Nutr Dieta. (1986) 38:173–81. doi: 10.1159/000412613 3516139

[16] Cha HY Yang SJ Kim S-W. Higher dietary inflammation in patients with schizophrenia: a case-control study in Korea. Nutrients. (2021) 13:2033. doi: 10.3390/nu13062033, 34199231 PMC8231973

[17] Suboticanec K Folnegović-Smalc V Turcin R Mestrović B Buzina R. Plasma levels and urinary vitamin C excretion in schizophrenic patients. Hum Nutr Clin Nutr. (1986) 40:421–8. 3793523

[18] Kim EJ Lim SY Lee HJ Lee J-Y Choi S Kim S-Y . Low dietary intake of n-3 fatty acids, niacin, folate, and vitamin C in Korean patients with schizophrenia and the development of dietary guidelines for schizophrenia. Nutr Res. (2017) 45:10–8. doi: 10.1016/j.nutres.2017.07.001, 29037327

[19] Fahira A Zhu Z Li Z Shi Y. Scrutinizing the causal relationship between schizophrenia and vitamin supplementation: a Mendelian randomization study. J Bio-X Res. (2022) 5:18–26. doi: 10.1097/JBR.0000000000000104, 42585464

[20] Kanofsky JD Sandyk R. Antioxidants in the treatment of schizophrenia. Int J Neurosci. (1991) 62:97–100. doi: 10.3109/00207459108999762, 1342020

[21] Sandyk R Kanofsky JD. Vitamin C in the treatment of schizophrenia. Int J Neurosci. (1993) 68:67–71. doi: 10.3109/00207459308994261 8063516

[22] Ramachandran P Thirunavakarasu P. Vitamins in schizophrenia: a literature review. Arch Ment Health. (2012) 13:74–9. Available online at: https://www.ovid.com/jnls/amhe/fulltext/02114888-201213020-00006~vitamins-in-schizophrenia-a-literature-review?an=02114888-201213020-00006

[23] Piłat P Nikel K Smolarczyk J Piegza M. The use of ascorbic acid in adjunctive treatment for schizophrenia—current state of knowledge. Life. (2024) 14:828. doi: 10.3390/life14070828, 39063582 PMC11278382

[24] Bošković M Vovk T. Can supplementation with vitamin E or C and omega-3 or −6 fatty acids improve the outcome of schizophrenia? Pharm J Slov. (2008) 59:167–73. Available online at: https://www.sfd.si/wp-content/uploads/2021/06/fv_4_2008_za_internet.pdf#page=5

[25] Firth J Stubbs B Sarris J Rosenbaum S Teasdale S Berk M . The effects of vitamin and mineral supplementation on symptoms of schizophrenia: a systematic review and meta-analysis. Psychol Med. (2017) 47:1515–27. doi: 10.1017/S0033291717000022, 28202095

[26] Brown HE Roffman JL. Vitamin supplementation in the treatment of schizophrenia. CNS Drugs. (2014) 28:611–22. doi: 10.1007/s40263-014-0172-4, 24846474 PMC4083629

[27] Magalhães PVS Dean O Andreazza AC Berk M Kapczinski F. Antioxidant treatments for schizophrenia. Cochrane Database Syst Rev. (2016) 2016:CD008919. doi: 10.1002/14651858.CD008919.pub2, 26848926 PMC10337514

[28] Yang M Wang C Zhao G Kong D Liu L Yuan S . Comparative analysis of the pre- and post-medication effects of antipsychotic agents on the blood-based oxidative stress biomarkers in patients with schizophrenia: a meta-analysis. Curr Neuropharmacol. (2023) 21:340–52. doi: 10.2174/1570159X20666220706101021, 35794775 PMC10190148

[29] Luchini C Stubbs B Solmi M Veronese N. Assessing the quality of studies in meta-analyses: advantages and limitations of the Newcastle Ottawa scale. World J Meta-Anal. (2017) 5:80. doi: 10.13105/wjma.v5.i4.80

[30] Slim K Nini E Forestier D Kwiatkowski F Panis Y Chipponi J. Methodological index for non-randomized studies (MINORS): development and validation of a new instrument. ANZ J Surg. (2003) 73:712–6. doi: 10.1046/j.1445-2197.2003.02748.x, 12956787

[31] McGuinness LA Higgins JPT. Risk-of-bias VISualization (robvis): an R package and shiny web app for visualizing risk-of-bias assessments. Res Synth Methods. (2021) 12:55–61. doi: 10.1002/jrsm.1411, 32336025

[32] Langan D Higgins JPT Jackson D Bowden J Veroniki AA Kontopantelis E . A comparison of heterogeneity variance estimators in simulated random-effects meta-analyses. Res Synth Methods. (2019) 10:83–98. doi: 10.1002/jrsm.1316, 30067315

[33] Dettori JR Norvell DC Chapman JR. Fixed-effect vs random-effects models for Meta-analysis: 3 points to consider. Global Spine J. (2022) 12:1624–6. doi: 10.1177/21925682221110527, 35723546 PMC9393987

[34] Love J Selker R Marsman M Jamil T Dropmann D Verhagen J . JASP: graphical statistical software for common statistical designs. J Stat Softw. (2019) 88:1–17. doi: 10.18637/jss.v088.i02, 33634423

[35] Myken AN Ebdrup BH Sørensen ME Broberg BV Skjerbæk MW Glenthøj BY . Lower vitamin C levels are associated with less improvement in negative symptoms in initially antipsychotic-naïve patients with first-episode psychosis. Int J Neuropsychopharmacol. (2022) 25:613–8. doi: 10.1093/ijnp/pyac029, 35532335 PMC9380709

[36] Shahini N Fotovat N Bagheri H Kashani L Bagheri Y. Micronutrient–immune interactions in mood and psychotic disorders: a case–control study of vitamin C, iron, zinc, magnesium, and peripheral blood cell indices. Sci Rep. (2026) 16:22359. doi: 10.1038/s41598-026-48616-x, 42144425 PMC13376511

[37] Bari BA Ivkovic A Wininger BA. Mild vitamin C deficiency is common in the inpatient psychiatric setting. J Clin Psychiatry. (2023) 84:22m14616. doi: 10.4088/JCP.22m14616, 37339367 PMC10428288

[38] Singh O Chakraborty I Dasgupta A Datta S. A comparative study of oxidative stress and interrelationship of important antioxidants in haloperidol and olanzapine treated patients suffering from schizophrenia. Indian J Psychiatry. (2008) 50:171–6. doi: 10.4103/0019-5545.43627, 19742187 PMC2738368

[39] Konarzewska B Stefańska E Wendołowicz A Cwalina U Golonko A Małus A . Visceral obesity in normal-weight patients suffering from chronic schizophrenia. BMC Psychiatry. (2014) 14:35. doi: 10.1186/1471-244X-14-35, 24506972 PMC3922935

[40] Strassnig M Singh Brar J Ganguli R. Dietary fatty acid and antioxidant intake in community-dwelling patients suffering from schizophrenia. Schizophr Res. (2005) 76:343–51. doi: 10.1016/j.schres.2005.03.002, 15949667

[41] Issa F Gerhardt GA Bartko JJ Suddath RL Lynch M Gamache PH . A multidimensional approach to analysis of cerebrospinal fluid biogenic amines in schizophrenia: I. Comparisons with healthy control subjects and neuroleptic-treated/unmedicated pairs analyses. Psychiatry Res. (1994) 52:237–49. doi: 10.1016/0165-1781(94)90069-8, 7991718

[42] Miljevlc C Nikolic A Damjanovlc A Salcic Z Spasic M Paunovlc V. Vitamin C and vitamin E in schizophrenic patients. Biol Psychiatry. (1997) 42:96S.

[43] Surapaneni KM. Status of lipid peroxidation, glutathione, ascorbic acid,vitamin E and antioxidant enzymes in schizophrenic patients. J Clin Diagn Res. (2007) 1:39–44., 17197733

[44] Subotičanec K Folnegović-Šmalc V Korbar M Meštrović B Buzina R. Vitamin C status in chronic schizophrenia. Biol Psychiatry. (1990) 28:959–66. doi: 10.1016/0006-3223(90)90061-6, 2275953

[45] Michael N Sourgens H Arolt V Erfurth A. Severe tardive dyskinesia in affective disorders: treatment with vitamin E and C. Neuropsychobiology. (2002) 46:28–30. doi: 10.1159/000068019, 12571430

[46] Pratiwi A Sukardi S Maliya A Sudiyanto A Muhlisin A Lestari T. Effect of relaxation therapy and vitamin C supplementation on stress and CD4 levels of mental illness patients. Biomed Pharmacol J. (2019) 12:423–9. doi: 10.13005/bpj/1656

[47] Ingole S Belorkar N Waradkar P Shrivastava M. Role of ascorbic acid supplementation on prevention of olanzapine induced metabolic side effects in schizophrenic patients. Indian J Public Health Res Dev. (2011) 2:12–6.

[48] Zhang J Chen S Chen J Zhang H Rao W-W. Comparison of olanzapine-induced weight gain and metabolism abnormalities between topiramate and vitamin C in patients with schizophrenia: a preliminary study. Front Psych. (2023) 14:1152953. doi: 10.3389/fpsyt.2023.1152953, 37252140 PMC10213308

[49] Vaughan K McConaghy N. Megavitamin and dietary treatment in schizophrenia: a randomised, controlled trial. Aust N Z J Psychiatry. (1999) 33:84–8. doi: 10.1046/j.1440-1614.1999.00527.x, 10197889

[50] Straw GM Bigelow LB Kirch DG. Haloperidol and reduced haloperidol concentrations and psychiatric ratings in schizophrenic patients treated with ascorbic acid. J Clin Psychopharmacol. (1989) 9:130–2. doi: 10.1097/00004714-198904000-00011, 2566628

[51] Sivrioglu EY Kirli S Sipahioglu D Gursoy B Sarandöl E. The impact of ω-3 fatty acids, vitamins E and C supplementation on treatment outcome and side effects in schizophrenia patients treated with haloperidol: an open-label pilot study. Prog Neuropsychopharmacol Biol Psychiatry. (2007) 31:1493–9. doi: 10.1016/j.pnpbp.2007.07.004, 17688987

[52] Bentsen H Osnes K Refsum H Solberg DK Bøhmer T. A randomized placebo-controlled trial of an omega-3 fatty acid and vitamins E+C in schizophrenia. Transl Psychiatry. (2013) 3:e335. doi: 10.1038/tp.2013.110, 24346133 PMC3906471

[53] Arvindakshan M Ghate M Ranjekar PK Evans DR Mahadik SP. Supplementation with a combination of ω-3 fatty acids and antioxidants (vitamins E and C) improves the outcome of schizophrenia. Schizophr Res. (2003) 62:195–204. doi: 10.1016/S0920-9964(02)00284-0, 12837515

[54] Beauclair L Vinogradov S Riney SJ Csernansky JG Hollister LE. An adjunctive role for ascorbic acid in the treatment of schizophrenia? J Clin Psychopharmacol. (1987) 7:282–3. doi: 10.1097/00004714-198708000-00037, 3624518

[55] Kausar S Khan S Ghani M Yousef N. Role of antioxidants in the management of schizophrenia: a randomized control trail. J Appl Mech Eng. (2021) 10:1–4.

[56] Dakhale GN Khanzode SD Khanzode SS Saoji A. Supplementation of vitamin C with atypical antipsychotics reduces oxidative stress and improves the outcome of schizophrenia. Psychopharmacology. (2005) 182:494–8. doi: 10.1007/s00213-005-0117-1, 16133138

[57] Tanra AJ Sabaruddin H Liaury K Zainuddin AA. Effect of adjuvant vitamin C on brain-derived neurotrophic factor levels and improvement of negative symptoms in schizophrenic patients. Open Access Maced J Med Sci. (2021) 9:353–7. doi: 10.3889/oamjms.2021.7086

[58] Zhang K Zou L Cai Y. Progress in the study of oxidative stress damage in patients with schizophrenia: challenges and opportunities. Front Psych. (2025) 16:1505397. doi: 10.3389/fpsyt.2025.1505397, 40060748 PMC11885282

[59] Bhatt S Upadhyay T Patil C Pai KSR Chellappan DK Dua K. Role of oxidative stress in pathophysiological progression of schizophrenia. Curr Psychiatry Res Rev. (2023) 19:11–27. doi: 10.2174/2666082218666220822154558

[60] May JM. Vitamin C transport and its role in the central nervous system. Subcell Biochem. (2012) 56:85–103. doi: 10.1007/978-94-007-2199-9_6 PMC3725125 22116696

[61] Bryll A Skrzypek J Krzyściak W Szelągowska M Śmierciak N Kozicz T . Oxidative-antioxidant imbalance and impaired glucose metabolism in schizophrenia. Biomolecules. (2020) 10:384. doi: 10.3390/biom10030384, 32121669 PMC7175146

[62] Cecerska-Heryć E Polikowska A Serwin N Roszak M Grygorcewicz B Heryć R . Importance of oxidative stress in the pathogenesis, diagnosis, and monitoring of patients with neuropsychiatric disorders, a review. Neurochem Int. (2022) 153:105269. doi: 10.1016/j.neuint.2021.105269, 34971747

[63] Dipasquale S Pariante CM Dazzan P Aguglia E McGuire P Mondelli V. The dietary pattern of patients with schizophrenia: a systematic review. J Psychiatr Res. (2013) 47:197–207. doi: 10.1016/j.jpsychires.2012.10.005, 23153955

[64] Anuradha A Kalekar MG Gadekar K. Oxidative stress, MDA, ascorbic acid, and vitamin E in newly diagnosed schizophrenia. Indian J Basic Appl Med Res. (2015) 4:260–4.

[65] Dadheech G Mishra S Gautam S Sharma P. Oxidative stress, α-tocopherol, ascorbic acid and reduced glutathione status in schizophrenics. Indian J Clin Biochem. (2006) 21:34–8. doi: 10.1007/BF02912908, 23105610 PMC3453982

[66] D’Souza B D’Souza V. Oxidative injury and antioxidant vitamins E and C in schizophrenia. Indian J Clin Biochem. (2003) 18:87–90. doi: 10.1007/BF02867671, 23105377 PMC3453727

[67] Ghodake S Suryakar A Padalkar R. The possible role of oxidants and antioxidant imbalance in pathophysiology of schizophrenia. Int J Med Res Heal Sci. (2012) 2:29–35.

[68] Issa AM Frhan HM Younis LA. Oxidative stress in schizophrenia. Kufa Med J. (2011) 14:41–6.

[69] Sankar DVS. Plasma levels of folates, riboflavin, vitamin B6, and ascorbate in severely disturbed children. J Autism Dev Disord. (1979) 9:73–82. doi: 10.1007/BF01531294, 438115

[70] Sarandol A Kirli S Akkaya C Altin A Demirci M Sarandol E. Oxidative–antioxidative systems and their relation with serum S100 B levels in patients with schizophrenia: effects of short term antipsychotic treatment. Prog Neuropsychopharmacol Biol Psychiatry. (2007) 31:1164–9. doi: 10.1016/j.pnpbp.2007.03.008, 17459548

[71] Toriumi K Iino K Ozawa A Miyashita M Yamasaki S Suzuki K . Glucuronic acid is a novel source of pentosidine, associated with schizophrenia. Redox Biol. (2023) 67:102876. doi: 10.1016/j.redox.2023.102876, 37703666 PMC10502438

[72] Uddin SMN Sultana F Uddin MG Dewan SMR Hossain MK Islam MS. Effect of antioxidant, malondialdehyde, macro-mineral, and trace element serum concentrations in Bangladeshi patients with schizophrenia: a case-control study. Health Sci Rep. (2021) 4:e291. doi: 10.1002/hsr2.291, 34013069 PMC8112814

[73] Young J McKinney SB Ross BM Wahle KWJ Boyle SP. Biomarkers of oxidative stress in schizophrenic and control subjects. Prostaglandins Leukot Essent Fatty Acids. (2007) 76:73–85. doi: 10.1016/j.plefa.2006.11.003, 17197163

[74] Khodaie M Alibeigi N Mirzaei VG. The effect of ascorbic acid as a supplementary treatment with risperidone in controlling the symptoms of schizophrenia: a double-blind, placebo-controlled clinical trial. J Basic Clin Pathophysiol. (2019) 7:7–14. doi: 10.22070/jbcp.2019.3616.1104

[75] Khan F Sultana S Mullick MSI Akhter N. Oxidative Stress and Antioxidant Status in Schizophrenia Patients. JAFMC Bangladesh (2016) 12:40–43.

[76] Rao YR P UD S M Khan MR. Status of Antioxidant Vitamins and Reduced Glutathione in Schizophrenia Patients with Positive, Negative and Cognitive Symptoms. World J Pharm Sci (2014) 2:528–533.

