# Supplementary material for: Examining the links between vitamin C and schizophrenia spectrum disorders: a systematic review and meta-analysis on circulating levels and supplementation outcomes
Source: Front Nutr. 2026 Oct 14;13:1965059. doi: 10.3389/fnut.2026.1965059 (PMC13713350; doi:10.3389/fnut.2026.1965059)
Supplement: Supplementary file 1 [file Supplementary_file_1.docx]

Supplementary Material

# 1 Supplementary Data Contents

**Supplementary Table 1**. PRISMA checklist

**Supplementary Table 2**. Characteristics of all studies eligible for the calculation of standardized mean difference in vitamin C levels between schizophrenia cases and controls

**Supplementary Table 3**. Characteristics of all studies eligible for the assessment of the effects of vitamin C supplementation on psychotic symptoms in schizophrenia cases

**Supplementary Data**

# SMD in vitC levels between SCZ cases and controls (includes Supplementary Figure 1 and Supplementary Tables 4 and 6)

- **SMD in psychotic symptom scores after versus before in SCZ cases under vitC supplementation** (includes **Supplementary Figure 2** and **Supplementary Tables 5** and **7**)

# 2 Supplementary Tables

**2.1	Supplementary Table 1. PRISMA checklist**

| **Section and Topic** | **Item #** | **Checklist item** | **Location where item is reported** |
| --- | --- | --- | --- |
| **TITLE** | | |  |
| Title | 1 | Identify the report as a systematic review. | Title |
| **ABSTRACT** | | |  |
| Abstract | 2 | See the PRISMA 2020 for Abstracts checklist. | Abstract |
| **INTRODUCTION** | | |  |
| Rationale | 3 | Describe the rationale for the review in the context of existing knowledge. | Introduction |
| Objectives | 4 | Provide an explicit statement of the objective(s) or question(s) the review addresses. | Introduction & section 2.1 |
| **METHODS** | | |  |
| Eligibility criteria | 5 | Specify the inclusion and exclusion criteria for the review and how studies were grouped for the syntheses. | Section 2.1 |
| Information sources | 6 | Specify all databases, registers, websites, organisations, reference lists and other sources searched or consulted to identify studies. Specify the date when each source was last searched or consulted. | Section 2.1 |
| Search strategy | 7 | Present the full search strategies for all databases, registers and websites, including any filters and limits used. | Section 2.1 |
| Selection process | 8 | Specify the methods used to decide whether a study met the inclusion criteria of the review, including how many reviewers screened each record and each report retrieved, whether they worked independently, and if applicable, details of automation tools used in the process. | Section 2.1 |
| Data collection process | 9 | Specify the methods used to collect data from reports, including how many reviewers collected data from each report, whether they worked independently, any processes for obtaining or confirming data from study investigators, and if applicable, details of automation tools used in the process. | Section 2.2 |
| Data items | 10a | List and define all outcomes for which data were sought. Specify whether all results that were compatible with each outcome domain in each study were sought (e.g. for all measures, time points, analyses), and if not, the methods used to decide which results to collect. | Section 2.2 & Tables 1-2 |
|  | 10b | List and define all other variables for which data were sought (e.g. participant and intervention characteristics, funding sources). Describe any assumptions made about any missing or unclear information. | Section 2.2 & Tables 1-2 |
| Study risk of bias assessment | 11 | Specify the methods used to assess risk of bias in the included studies, including details of the tool(s) used, how many reviewers assessed each study and whether they worked independently, and if applicable, details of automation tools used in the process. | Section 2.2 |
| Effect measures | 12 | Specify for each outcome the effect measure(s) (e.g. risk ratio, mean difference) used in the synthesis or presentation of results. | Section 2.3 |
| Synthesis methods | 13a | Describe the processes used to decide which studies were eligible for each synthesis (e.g. tabulating the study intervention characteristics and comparing against the planned groups for each synthesis (item #5)). | Section 2.1, Tables 1-2 & Section 3.1 |
|  | 13b | Describe any methods required to prepare the data for presentation or synthesis, such as handling of missing summary statistics, or data conversions. | Sections 2.2 & Tables 1-2 |
|  | 13c | Describe any methods used to tabulate or visually display results of individual studies and syntheses. | Tables 1-2 |
|  | 13d | Describe any methods used to synthesize results and provide a rationale for the choice(s). If meta-analysis was performed, describe the model(s), method(s) to identify the presence and extent of statistical heterogeneity, and software package(s) used. | Section 2.3 |
|  | 13e | Describe any methods used to explore possible causes of heterogeneity among study results (e.g. subgroup analysis, meta-regression). | Sections 2.3, 2.4 and 2.5 |
|  | 13f | Describe any sensitivity analyses conducted to assess robustness of the synthesized results. | Section 2.4 |
| Reporting bias assessment | 14 | Describe any methods used to assess risk of bias due to missing results in a synthesis (arising from reporting biases). | Section 2.5 |
| Certainty assessment | 15 | Describe any methods used to assess certainty (or confidence) in the body of evidence for an outcome. | Section 2.5 |
| **RESULTS** | | |  |
| Study selection | 16a | Describe the results of the search and selection process, from the number of records identified in the search to the number of studies included in the review, ideally using a flow diagram. | Section 3.1 & Figure 1 |
|  | 16b | Cite studies that might appear to meet the inclusion criteria, but which were excluded, and explain why they were excluded. | Section 3.1 |
| Study characteristics | 17 | Cite each included study and present its characteristics. | Tables 1-2 |
| Risk of bias in studies | 18 | Present assessments of risk of bias for each included study. | Sections 3.2-3.3 & Figures 2-3 |
| Results of individual studies | 19 | For all outcomes, present, for each study: (a) summary statistics for each group (where appropriate) and (b) an effect estimate and its precision (e.g. confidence/credible interval), ideally using structured tables or plots. | Sections 3.2 and 3.3 & Figures 4-5 |
| Results of syntheses | 20a | For each synthesis, briefly summarise the characteristics and risk of bias among contributing studies. | Sections 3.2 and 3.3 & Figures 4-5 |
|  | 20b | Present results of all statistical syntheses conducted. If meta-analysis was done, present for each the summary estimate and its precision (e.g. confidence/credible interval) and measures of statistical heterogeneity. If comparing groups, describe the direction of the effect. | Sections 3.2 and 3.3 & Figures 4-5 |
|  | 20c | Present results of all investigations of possible causes of heterogeneity among study results. | Sections 3.2 and 3.3 & Tables 3-4 |
|  | 20d | Present results of all sensitivity analyses conducted to assess the robustness of the synthesized results. | Sections 3.2 and 3.3 & Tables 3-4 |
| Reporting biases | 21 | Present assessments of risk of bias due to missing results (arising from reporting biases) for each synthesis assessed. | Sections 3.2 and 3.3 & Figures 4-5 |
| Certainty of evidence | 22 | Present assessments of certainty (or confidence) in the body of evidence for each outcome assessed. | Sections 3.2 and 3.3 |
| **DISCUSSION** | | |  |
| Discussion | 23a | Provide a general interpretation of the results in the context of other evidence. | Discussion |
|  | 23b | Discuss any limitations of the evidence included in the review. | Discussion |
|  | 23c | Discuss any limitations of the review processes used. | Discussion |
|  | 23d | Discuss implications of the results for practice, policy, and future research. | Discussion |
| **OTHER INFORMATION** | | |  |
| Registration and protocol | 24a | Provide registration information for the review, including register name and registration number, or state that the review was not registered. | N/A |
|  | 24b | Indicate where the review protocol can be accessed, or state that a protocol was not prepared. | N/A |
|  | 24c | Describe and explain any amendments to information provided at registration or in the protocol. | N/A |
| Support | 25 | Describe sources of financial or non-financial support for the review, and the role of the funders or sponsors in the review. | Acknowledgments |
| Competing interests | 26 | Declare any competing interests of review authors. | Declaration of interests |
| Availability of data, code and other materials | 27 | Report which of the following are publicly available and where they can be found: template data collection forms; data extracted from included studies; data used for all analyses; analytic code; any other materials used in the review. | Tables 1-2 |

*From:* Page MJ, McKenzie JE, Bossuyt PM, Boutron I, Hoffmann TC, Mulrow CD, et al. The PRISMA 2020 statement: an updated guideline for reporting systematic reviews. BMJ 2021;372:n71. doi: 10.1136/bmj.n71. This work is licensed under CC BY 4.0. To view a copy of this license, visit <https://creativecommons.org/licenses/by/4.0/>

## 2.2 Supplementary Table 2. Characteristics of all studies eligible for the calculation of standardized mean difference (SMD) in vitamin C levels between SCZ cases and controls

| **S. No.** | **First author (Year) (Reference)** | **Country of study** | **Study type** | **SCZ diagnostic instrument** | **SCZ cases characteristics** | **Ctrl characteristics** | **Sample size** | | **Age (mean** ± **SD)** | | **VitC measurement instrument (sample used)** | **VitC levels (mean ± SD)** | | |
| --- | --- | --- | --- | --- | --- | --- | --- | --- | --- | --- | --- | --- | --- | --- |
|  |  |  |  |  |  |  | **Ctrl** | **SCZ** | **Ctrl** | **SCZ** |  | **Ctrl** | **SCZ** |  |
| 1 | Anuradha (2015) (1) | India | Case-control | DSM‑IV | New diagnosed SCZ cases under no medication for at least 2 weeks | Age and gender matched healthy controls | 30 | 90 | 30.20 ± 5.4 | | Spectrophotometry (serum) | 1.19 ± 0.14 mg/dL | 1.04 ± 0.14 mg/dL^$^ |  |
| 2 | Dadheech (2006) (2) | India | Case-control | Clinical symptomatic diagnosis and PANSS scores | Newly diagnosed acute and chronic SCZ outpatients | Age and gender matched healthy controls | 40 | 58 | NS | NS | Spectrophotometry (serum) | 1.26 ± 0.43 mg/dL | 0.75 ± 0.21 mg/dL^$^ |  |
| 3 | D’Souza (2003) (3) | India | Case-control | Clinical symptomatic diagnosis | Chronic SCZ inpatients under prescribed medication | Age and gender matched normal subjects | 18 | 14 | ND | 29-68 | Spectrophotometry (plasma) | 0.8078 ± 0.51965 mg/dL^#^ | 0.2904 ± 0.196 mg/dL^#$^ |  |
| 4 | Ghodake (2012) (4) | India | Case-control | ICD‑10 | SCZ in- and outpatients under no medication for at least 2 weeks | Age and gender matched healthy volunteers | 30 | 30 | 37.2 ± 13.8 | 35.50 ± 9.4 | Spectrophotometry (plasma) | 0.90 ± 0.21 mg/dL | 0.63 ± 0.14 mg/dL^$^ |  |
| 5 | Issa (2011) (5) | Iraq | Case-control | DSM-IV | SCZ outpatients | Healthy subjects | 30 | 70 | 15-60 | 18-65 | Spectrophotometry (serum) | 3.41 ± 0.83 mg/dL | 1.75 ± 1.01 mg/dL^$^ |  |
| 6 | Khan (2016) (6) | Bangladesh | Case-control | DSM‑V | Adult SCZ in- and outpatients with no antipsychotic medication for last 30 days | Age and sex matched “apparently” healthy individuals | 30 | 93 | ND | ≥18 | Spectrophotometry (plasma) | 78.7 ± 10.9 μmol/L  (1.39 ± 0.19 mg/dL) | 30.6 ± 8.3 μmol/L  0.54 ± 0.15 (mg/dL)^$^ |  |
| 7 | Rao (2014) (7) | India | Case-control | DSM‑IV | SCZ inpatients with “good socio-economic background” and under a standard diet | Age and sex matched healthy normal controls with no individual and familial history of mental illnesses | 60 | 60 (20 subjects each with positive, negative, and cognitive symptoms) | 15-65 | 18-65 | Spectrophotometry (plasma) | 1.78 ± 0.13 mg/dL | Positive: 0.54 ± 0.18 mg/dL^$^ (n=20)  Negative: 0.95 ± 0.12 mg/dL^$^ (n=20)  Cognitive: 1.04 ± 0.17 mg/dL^$^ (n=20)  Combined: 0.8433 ± 0.2693 mg/dL |  |
| 8 | Sankar (1979)* (8) | USA | Case-control | Clinical “psychometric, psychological, and psychiatric” diagnosis | Childhood SCZ cases under no medication | Children with other psychiatric disorders | Behavioural disorders: 6  Other psychoses: 13  Mental deficiency: 5  Combined: 24 | SCZ (non-autistic): 78  SCZ (autistic): 19  Combined: 97 | 5-16 | 5-16 | Spectrophotometry (plasma) | Behavioural disorders: 950 ± 350 μg/100 mL  Other psychoses: 1060 ± 320 μg/100 mL  Mental deficiency: 1120 ± 280 μg/100 mL  Combined: 1045 ± 312.0828 μg/100 ml (1.045 ± 0.312 mg/dL) | SCZ (non-autistic): 960 ± 370 μg/100 mL  SCZ (autistic): 760 ± 460 μg/100 mL  Combined:  920.8247 ± 394.7736 μg/100 ml (0.9208 ± 0.3948 mg/dL) |  |
| 9 | Sarandol (2007) (9) | Turkey | Case-control | DSM‑IV | SCZ in- and outpatients either drug-naïve or antipsychotic-free for at least 3 weeks | Age, gender and smoking status matched healthy controls | 35 | 40 | 33.5 ± 9.2 | 34.9 ± 9.9 | Spectrophotometry (plasma) | 55.8 ± 26.1 μmol/L^##^ (0.983 ± 0.460 mg/dL) | 51.7 ± 35.6 μmol/L^##^ (0.910 ± 0.627 mg/dL) |  |
| 10 | Subotičanec (1990)* (10) | Former Yugoslavia (Croatia) | Case-control | ICD | Inpatients | Cases with somatoform or anxiety disorders recruited from the same hospital | 35 | 35 | 37-49 | 37-49 | Fluorimetry (plasma) | Group 1: 0.63 ± 0.44 mg/dL (n = 15)  Group 2: 0.73 ± 0.55 mg/dL (n = 20)  Combined: 0.6871 ± 0.5013 mg/dL | Group 1: 0.36 ± 0.27 mg/dL (n = 15)  Group 2: 0.41 ± 0.36 mg/dL (n = 20)  Combined: 0.3886 ± 0.321 mg/dL |  |
| 11 | Surapaneni (2007) (11) | India | Case-control | DSM‑IV | Newly diagnosed SCZ outpatients | Age and sex matched healthy controls | 48 | 48 | NS | 22-65 | Spectrophotometry (plasma) | 11.88 ± 0.22 mg/dL | 11.36 ± 0.11 mg/dL^$^**^** |  |
| 12 | Toriumi (2023) (12)^&^ | Japan | Case-control | DSM-IV | SCZ in- and outpatients | Healthy controls | Cohort 1: 50  Cohort 2: 67  Combined: 117 | Cohort 1: 98  Cohort 2: 120  Combined: 218 | NS | NS | Mass spectrometry (plasma) | Cohort 1: 0.93178944 ± 1.247113276  Cohort 2: 1.765427104 ± 1.4441156  Combined: 1.4092 ± 1.4195 AU | Cohort 1: 0.887372643 ± 1.369723607  Cohort 2: 1.201444858 ± 1.752290786  Combined: 1.0603 ± 1.5959 AU |  |
| 13 | Uddin (2021) (13) | Bangladesh | Case-control | DSM‑IV‑TR | SCZ cases | Age matched healthy volunteers | 63 | 63 | 27.74 ± 0.93 | 29.28 ± 1.19 | Spectrophotometry (serum) | 11.08 ± 0.31 μmol/L (mean ± SEM)  OR  11.08 ± 2.46 μmol/L (mean ± SD)  (0.195 ± 0.043 mg/dL) | 7.55 ± 0.43 μmol/L (mean ± SEM)  OR  7.55 ± 3.41 μmol/L (mean ± SD)  (0.133 ± 0.060 mg/dL)^$^ |  |
| 14 | Young (2007) (14) | UK | Case-control | DSM‑IV | SCZ inpatients under different medicinal regimens | Age, gender and smoking status matched “apparently” healthy controls | 17 | 16 | 38.9 ± 9.2 | 37.9 ± 11.0 | RP HPLC (plasma) | 54.56 ± 1.28 μM (mean ± SEM)  OR  54.56 ± 5.28 μM (mean ± SD)  (0.961 ± 0.093 mg/dL) | 45.70 ± 0.98 μM  OR  45.70 ± 3.92 μM (mean ± SD)  (0.805 ± 0.069 mg/dL) |  |

* denotes Ctrl group was patients with other neurological illnesses, ^&^ denotes data extracted from supplementary information, ^#^ denotes probable typographical error in units reported in the primary study (given units: mg/L; likely units: mg/dL), ^##^ denotes possible typographical error in units reported in the primary study (given units: mmol/L; likely units: μmol/L), ^$^ denotes statistically significant differences in vitC levels between SCZ and Ctrl subjects as determined by the authors of the primary study, **^** denotes physiologically implausible levels of vitC (hence the study was excluded).

*Abbreviations:* AU, arbitrary units; DSM, Diagnostic and Statistical Manual of Mental Disorders; HPLC-CEAS, high-performance liquid chromatography with coulometric electrochemical array sensor/detection; ICD, Diagnostic and Statistical Manual of Mental Disorders; PANSS, Positive and Negative Syndrome Scale; NS, not stated; RP HPLC, reverse phase- high-performance liquid chromatography.

**2.3	Supplementary Table 3. Characteristics of all studies eligible for the assessment of the effects of vitamin C supplementation on psychotic symptoms in SCZ cases**

| **S. No.** | **First author (Year) (Reference)** | **Country** | **Study type** | **SCZ diagnostic instrument** | **SCZ cases characteristics (age)** | **Sample size** | | | **VitC dose and duration** | **Disease outcome scoring instrument** | **Outcome scores in placebo/control group (mean ± SD)** | | **Outcome scores in vitC supplemented group (mean ± SD)** | |
| --- | --- | --- | --- | --- | --- | --- | --- | --- | --- | --- | --- | --- | --- | --- |
|  |  |  |  |  |  | **Placebo/control** | **VitC** |  | |  | **Baseline** | **Final** | **Baseline** | **Final** |
| 1 | Arvindakshan (2003) (15)* | India | Single arm pre-post-trial | DSM-IV | SCZ cases on antipsychotic medication (29.57 ± 7.03 yr) | NR | 28 | 2X500 mg/day for 4 months | | PANSS | NR | NR | 80 ± 26.9 | 61 ± 22.7 |
|  |  |  |  |  |  |  |  |  |  | BPRS |  |  | 45 ± 16.5 | 35 ± 13.7 |
| 2 | Beauclair (1987) (16) | USA | Single arm pre-post-trial | NS | SCZ cases stabilized under medication (NS) | NR | 13 | Escalating doses for a period of 8 weeks: 1 g/day (weeks 1-2), 2 g/day (weeks 3-4), 4 g/day (weeks 5-6), 8 g/day (weeks 7-8) | | BPRS | NR | NR | Mean=42; range = 34-52  (42 ± 4.5) | Mean=34; range = 26-48  (34 ± 5.5) |
| 3 | Dakhale (2005) (17) | India | Randomized, double-blind, placebo-controlled, pre-post-trial | DSM-IV | SCZ outpatients on atypical antipsychotic medication (36.3 ± 12.8 and 40.8 ± 10.2 yr in placebo and vitC groups) | 20 | 20 | 500 mg/day for 8 weeks | | BPRS | 35.90 ± 5.41 | 28.96 ± 6.16^$^ | 36.65 ± 5.78 | 19.30 ± 5.46^$^ |
| 4 | Khodaie (2019) (18) | Iran | Randomized, double-blind, placebo-controlled, pre-post-trial | DSM-V | SCZ inpatients under risperidone treatment (18-45 yr) | 22 | 22 | 2X500 mg/day for 4 months | | PANSS | 82.3 ± 15.9 | 73.5 ± 16.9 | 86.3 ± 18.9 | 65.1 ± 18.7^$^ |
| 5 | Kausar (2021) (19) | Pakistan | Randomized, placebo-controlled, pre-post-trial | DSM-IV-TR | SCZ cases (20-50 yr) | 35 | 35 | 500 mg/day for 8 weeks | | PANSS | NS | NS | 88.72 ± 18.39 | 46.93 ± 8.22 |
| 6 | Tanra (2021) (20) | Indonesia | Controlled, pre-post-trial | DSM-V | SCZ cases (18-50 yr) under atypical antipsychotic therapy | 30 | 27 | 1000 mg/day for | | PANSS (TSS)^&^ | 39.07 ± 5.199 | 27.20 ± 4.773 | 38.74 ± 5.281 | 13.19 ± 4.860^$^ |

* denotes supplementation of vitC was performed along with other dietary therapeutics (e.g., fatty acids and/or vitamin E), ^$^ denotes statistically significant differences in pre and post symptom scores as determined by the authors of the primary study, ^&^ denotes only total negative symptom score reported.

Abbreviations: BPRS, Brief Psychiatric Rating Scale; DSM, Diagnostic and Statistical Manual of Mental Disorders; PANSS, Positive and Negative Syndrome Scale; NR, not recruited; NS, not stated; NSS, positive symptom score (PANSS subdomain)

**3. Supplementary data**

**3.1. SMD in vitC levels between SCZ cases and controls**

Thirteen case-control studies (**Supplementary Table 4**), enrolling a total of 884 SCZ subjects and 529 controls, were eligible for evaluating the differences in the circulating levels of vitC. The pooled SMD (SCZ minus controls) was estimated at -1.628 [95% CI: -2.481, -0.775], and this was statistically significant (Z=-3.74, p<0.001; **Supplementary** **Figure 1A**). Significant heterogeneity amongst contributing studies was detected by Cochran's Q analysis (Q=305.51, dof=12, p<0.001), with the τ^2^ and I^2^ statistics estimated to be 2.286 and 97.25%, respectively. Egger’s regression test (t=-2.388, p=0.036) indicated significant presence of publication bias. However, Begg’s rank correlation test (τ=-0.410, p=0.057) failed to identify significant publication bias. Correspondingly, Duval and Tweedie's trim and fill method did not find any hypothetical studies missed due to small-study effects (**Supplementary** **Figure 1B**). Rosenthal’s fail-safe N estimate of 1482 indicated the requirement of 1482 null studies to render the overall effect non‑significant, further confirming the robustness of the results. Rosenberg’s approach yielded a similarly large fail-safe N of 714, again supporting the stability of the results. Lastly, case-wise sensitivity diagnostics indicated Khan (2016) and Rao (2014) to be potentially influential studies (**Supplementary** **Figure 1C, Supplementary Table 5**).

**Supplementary Table 4. List of studies evaluating the differences in vitC levels between SCZ cases and controls**

| **S. No.** | **First author (Year) (Reference)** | **Country of study** | **Sample size** | | | **VitC levels**  **(mg/dL; mean ± SD)** | |
| --- | --- | --- | --- | --- | --- | --- | --- |
|  |  |  | **Ctrl** | **SCZ** | **Ctrl** | | **SCZ** |
| 1 | Anuradha (2015) (1) | India | 30 | 90 | 1.19 ± 0.14 | | 1.04 ± 0.14 |
| 2 | Dadheech (2006) (2) | India | 40 | 58 | 1.26 ± 0.43 | | 0.75 ± 0.21 |
| 3 | D’Souza (2003) (3) | India | 18 | 14 | 0.8078 ± 0.51965 | | 0.2904 ± 0.196 |
| 4 | Ghodake (2012) (4) | India | 30 | 30 | 0.90 ± 0.21 | | 0.63 ± 0.14 |
| 5 | Issa (2011) (5) | Iraq | 30 | 70 | 3.41 ± 0.83 | | 1.75 ± 1.01 |
| 6 | Khan (2016) (6) | Bangladesh | 30 | 93 | 1.39 ± 0.19 | | 0.54 ± 0.15 |
| 7 | Rao (2014) (7) | India | 60 | 60 | 1.78 ± 0.13 | | 0.8433 ± 0.2693 |
| 8 | Sankar (1979) (8) | USA | 24 | 97 | 1.045 ± 0.312 | | 0.9208 ± 0.3948 |
| 9 | Sarandol (2007) (9) | Turkey | 35 | 40 | 0.983 ± 0.460 | | 0.910 ± 0.627 |
| 10 | Subotičanec (1990) (10) | Former Yugoslavia (Croatia) | 35 | 35 | 0.6871 ± 0.5013 | | 0.3886 ± 0.321 |
| 11 | Toriumi (2023) (12) | Japan | 117 | 218 | 1.4092 ± 1.4195 | | 1.0603 ± 1.5959 |
| 12 | Uddin (2021) (13) | Bangladesh | 63 | 63 | 0.195 ± 0.043 | | 0.133 ± 0.060 |
| 13 | Young (2007) (14) | UK | 17 | 16 | 0.961 ± 0.093 | | 0.805 ± 0.069 |

**Supplementary Figure 1.** Meta-analytical assessment of the differences in the circulating vitC levels in SCZ cases versus controls. (A) Forest plot shows a significant reduction in vitC levels in SCZ cases, as indicated by the pooled SMD. (B) Funnel plot shows largely negative distribution of the point estimates across studies, with no potentially missed studies identified in the trim and fill test. (C) Baujat plot identifies two studies (Khan [2016] and Rao [2014]) with disproportionately high influences on the pooled effect estimate.

**Supplementary Table 5. Case-wise diagnostics table for studies evaluating vitC level in SCZ subjects versus controls**

| **First author (Year) (Reference)** | **Standardized Residual** | **DFFITS** | **Cook's Distance** | **Covariance ratio** | **Leave-one-out** | | **Influential** |
| --- | --- | --- | --- | --- | --- | --- | --- |
|  |  |  |  |  | **𝜏** | **Qₑ** |  |
| Anuradha (2015) | 0.368 | 0.104 | 0.012 | 1.174 | 1.576 | 305.4 | No |
| Dadheech (2006) | 0.025 | 0.007 | 5.377 x10^-5^ | 1.187 | 1.585 | 298.3 | No |
| D’Souza (2003) | 0.258 | 0.074 | 0.006 | 1.174 | 1.578 | 305.1 | No |
| Ghodake (2012) | 0.087 | 0.025 | 6.674 x10^-4^ | 1.184 | 1.584 | 302.3 | No |
| Issa (2011) | -0.057 | -0.016 | 2.798 x10^-4^ | 1.186 | 1.584 | 296.3 | No |
| **Khan (2016)** | -3.221 | -0.924 | 0.485 | 0.615 | 1.105 | 185.2 | **Yes** |
| Rao (2014) | -2.134 | -0.607 | 0.282 | 0.832 | 1.308 | 198.9 | No |
| Sankar (1979) | 0.875 | 0.247 | 0.062 | 1.113 | 1.531 | 296.4 | No |
| Sarandol (2007) | 1.017 | 0.287 | 0.082 | 1.088 | 1.512 | 290.7 | No |
| Subotičanec (1990) | 0.610 | 0.173 | 0.032 | 1.150 | 1.558 | 304.1 | No |
| Toriumi (2023) | 0.954 | 0.267 | 0.072 | 1.100 | 1.520 | 239.5 | No |
| Uddin (2021) | 0.277 | 0.083 | 0.007 | 1.158 | 1.572 | 305.4 | No |
| Young (2007) | -0.125 | -0.041 | 0.002 | 1.121 | 1.563 | 304.8 | No |

Abbreviations: DFFITS, difference in fits.

**3.2. SMD in psychotic symptom scores after versus before in SCZ cases under vitC supplementation**

For the evaluation of the psychopathological improvements amongst SCZ cases upon vitC supplementation, six studies with seven datasets, recruiting a cumulative of 145 SCZ cases (**Supplementary Table 6**), were identified. Between-group differences (post-supplementation minus baseline) in psychopathological scores were found to be significant, as indicated by the pooled SMD of -2.094 [95% CI: -3.235, -0.953] (Z=-3.60, p<0.001; **Supplementary** **Figure 2A**). Cochran’s assessment identified significant inter-study heterogeneity (Q=85.04, dof=6, p<0.001); and the τ^2^ and I^2^ statistics were calculated to be 2.219 and 94.62%, respectively. Both Egger’s (t=-3.332, p=0.021) and Begg’s (τ=-0.810, p=0.011) tests predicted significant publication bias. However, trim and fill analysis failed to identify any potential study which could have been missed due to publication bias (**Supplementary** **Figure 2B**). Rosenthal’s and Rosenberg’s fail-safe N values were estimated at 472 and 262, indicating general robustness of the pooled summary measure. Lastly, sensitivity analysis represented as case-wise sensitivity diagnostic measures pointed to Tanra (2021) as an influential study with disproportionately large contribution to the pooled point estimate (**Supplementary** **Figure 2C, Supplementary** **Table 7**).

**Supplementary Table 6. List of trials examining the effects of vitC supplementation in SCZ cases**

| **S. No.** | **First author (Year) (Reference)** | **Country** | **Study type** | **Sample size** | **VitC dose and duration** | **Disease outcome scoring instrument** | **Baseline total psychotic symptom score (mean ± SD)** | **Final total psychotic symptom score (mean ± SD)** |
| --- | --- | --- | --- | --- | --- | --- | --- | --- |
| 1 | Arvindakshan (2003) (15) | India | Single arm pre-post-trial | 28 | 2 x 500 mg/day for 4 months | PANSS (TS) | 80 ± 26.9 | 61 ± 22.7 |
|  |  |  |  |  |  | BPRS | 45 ± 16.5 | 35 ± 13.7 |
| 2 | Beauclair (1987) (16) | USA | Single arm pre-post-trial | 13 | Escalating doses for a period of 8 weeks: 1 g/day (weeks 1-2), 2 g/day (weeks 3-4), 4 g/day (weeks 5-6), 8 g/day (weeks 7-8) | BPRS | 42 ± 4.5 | 34 ± 5.5 |
| 3 | Dakhale (2005) (17) | India | Randomized, double-blind, placebo-controlled, pre-post-trial | 20 | 500 mg/day for 8 weeks | BPRS | 36.65 ± 5.78 | 19.30 ± 5.46 |
| 4 | Khodaie (2019) (18) | Iran | Randomized, double-blind, placebo-controlled, pre-post-trial | 22 | 2 x 500 mg/day for 4 months | PANSS (TS) | 86.3 ± 18.9 | 65.1 ± 18.7 |
| 5 | Kausar (2021) (19) | Pakistan | Randomized, placebo-controlled, pre-post-trial | 35 | 500 mg/day for 8 weeks | PANSS (TS) | 88.72 ± 18.39 | 46.93 ± 8.22 |
| 6 | Tanra (2021) (20) | Indonesia | Controlled, pre-post-trial | 27 | 1000 mg/day for 8 weeks | PANSS (NSS) | 38.74 ± 5.281 | 13.19 ± 4.860^$^ |

Abbreviations: BPRS, Brief Psychiatric Rating Scale; NSS, negative symptom score; PANSS, Positive and Negative Syndrome Scale; TS, total score.

**Supplementary Figure 2.** Meta-analytical assessment of the changes in SCZ psychopathology upon exogenous vitC supplementation. (A) Forest plot shows a significant improvement in psychotic scores after vitC supplementation in SCZ cases, compared to baseline scores. (B) Funnel plot shows consistent negative point estimates across studies; no potential studies were identified to be missed due to small-study effects by the trim and fill analysis. (C) Baujat plot indicates more-or-less uniform distribution of studies, with only Tanra [2021] identified as having potentially outlying influences on the pooled summary measure.

**Supplementary Table 7. Case-wise diagnostics table for vitC supplementation trials**

| **First author (Year) (Reference)** | **Standardized Residual** | **DFFITS** | **Cook's Distance** | **Covariance ratio** | **Leave-one-out** | | **Influential** |
| --- | --- | --- | --- | --- | --- | --- | --- |
|  |  |  |  |  | **𝜏** | **Qₑ** |  |
| Arvindakshan (2003) TS-PANSS | 0.946 | 0.395 | 0.160 | 1.204 | 1.511 | 72.80 | No |
| Arvindakshan (2003) BPRS | 1.034 | 0.430 | 0.184 | 1.168 | 1.486 | 69.33 | No |
| Beauclair (1987) BPRS | 0.357 | 0.154 | 0.028 | 1.372 | 1.626 | 85.02 | No |
| Dakhale (2005) BPRS | -0.607 | -0.238 | 0.063 | 1.296 | 1.578 | 74.78 | No |
| Khodaie (2019) TS-PANSS | 0.665 | 0.281 | 0.088 | 1.305 | 1.579 | 82.23 | No |
| Kausar (2021) TS-PANSS | -0.532 | -0.211 | 0.051 | 1.331 | 1.596 | 68.16 | No |
| **Tanra (2021) NSS-PANSS** | -2.753 | -1.114 | 0.629 | 0.544 | 0.988 | 45.41 | **Yes** |

Abbreviations: DFFITS, difference in fits.

**4.	References**

1.	Anuradha A, Kalekar MG, Gadekar K. Oxidative stress, MDA, ascorbic acid, and vitamin E in newly diagnosed schizophrenia. *Indian J Basic Appl Med Res* (2015) 4:260–264.

2.	Dadheech G, Mishra S, Gautam S, Sharma P. Oxidative stress, α-tocopherol, ascorbic acid and reduced glutathione status in schizophrenics. *Indian J Clin Biochem* (2006) 21:34–38. doi: 10.1007/BF02912908

3.	D’Souza B, D’Souza V. Oxidative injury and antioxidant vitamins E and C in Schizophrenia. *Indian J Clin Biochem* (2003) 18:87–90. doi: 10.1007/BF02867671

4.	Ghodake S, Suryakar A, Padalkar R. The possible role of oxidants and antioxidant imbalance in pathophysiology of schizophrenia. *Int J Med Res Heal Sci* (2012) 2:29–35.

5.	Issa AM, Frhan HM, Younis LA. Oxidative stress in schizophrenia. *Kufa Med J* (2011) 14:41–46.

6.	Khan F, Sultana S, Mullick M, N A. Oxidative Stress and Antioxidant Status in Schizophrenia Patients. *JAFMC Bangladesh* (2016) 12:40–43.

7.	Rao YR, P UD, S M, Khan MR. Status of Antioxidant Vitamins and Reduced Glutathione in Schizophrenia Patients with Positive, Negative and Cognitive Symptoms. *World J Pharm Sci* (2014) 2:528–533.

8.	Sankar DVS. Plasma levels of folates, riboflavin, vitamin B6, and ascorbate in severely disturbed children. *J Autism Dev Disord* (1979) 9:73–82. doi: 10.1007/BF01531294

9.	Sarandol A, Kirli S, Akkaya C, Altin A, Demirci M, Sarandol E. Oxidative–antioxidative systems and their relation with serum S100 B levels in patients with schizophrenia: Effects of short term antipsychotic treatment. *Prog Neuro-Psychopharmacology Biol Psychiatry* (2007) 31:1164–1169. doi: 10.1016/j.pnpbp.2007.03.008

10.	Subotičanec K, Folnegović-Šmalc V, Korbar M, Meštrović B, Buzina R. Vitamin C status in chronic schizophrenia. *Biol Psychiatry* (1990) 28:959–966. doi: 10.1016/0006-3223(90)90061-6

11.	Surapaneni KM, Venkataramana G. Status of lipid peroxidation, glutathione, ascorbic acid, vitamin E and antioxidant enzymes in patients with osteoarthritis. *Indian J Med Sci* (2007) 61:9–14. http://www.ncbi.nlm.nih.gov/pubmed/17197733

12.	Toriumi K, Iino K, Ozawa A, Miyashita M, Yamasaki S, Suzuki K, Sugawa H, Tabata K, Yamaguchi S, Usami S, et al. Glucuronic acid is a novel source of pentosidine, associated with schizophrenia. *Redox Biol* (2023) 67:102876. doi: 10.1016/j.redox.2023.102876

13.	Uddin SMN, Sultana F, Uddin MG, Dewan SMR, Hossain MK, Islam MS. Effect of antioxidant, malondialdehyde, macro‐mineral, and trace element serum concentrations in Bangladeshi patients with schizophrenia: A case‐control study. *Heal Sci Reports* (2021) 4:e291. doi: 10.1002/hsr2.291

14.	Young J, McKinney SB, Ross BM, Wahle KWJ, Boyle SP. Biomarkers of oxidative stress in schizophrenic and control subjects. *Prostaglandins, Leukot Essent Fat Acids* (2007) 76:73–85. doi: 10.1016/j.plefa.2006.11.003

15.	Arvindakshan M. Supplementation with a combination of ω-3 fatty acids and antioxidants (vitamins E and C) improves the outcome of schizophrenia. *Schizophr Res* (2003) 62:195–204. doi: 10.1016/S0920-9964(02)00284-0

16.	Beauclair L, Vinogradov S, Riney SJ, Csernansky JG, Hollister LE. An Adjunctive Role for Ascorbic Acid in the Treatment of Schizophrenia? *J Clin Psychopharmacol* (1987) 7:282. doi: 10.1097/00004714-198708000-00037

17.	Dakhale GN, Khanzode SD, Khanzode SS, Saoji A. Supplementation of vitamin C with atypical antipsychotics reduces oxidative stress and improves the outcome of schizophrenia. *Psychopharmacology (Berl)* (2005) 182:494–498. doi: 10.1007/s00213-005-0117-1

18.	Khodaie M, Alibeigi N, Mirzaei VG. The effect of ascorbic acid as a supplementary treatment with risperidone in controlling the symptoms of schizophrenia: A double-blind, placebo-controlled clinical trial. *J Basic Clin Pathophysiol* (2019) 7:7–14. doi: 10.22070/jbcp.2019.3616.1104

19.	Kausar S, Khan S, Ghani M, Yousef N. Role of antioxidants in the management of Schizophrenia: a Randomized Control Trail. *J Appl Mech Eng* (2021) 10:1–4.

20.	Tanra AJ, Sabaruddin H, Liaury K, Zainuddin AA. Effect of Adjuvant Vitamin C on Brain-Derived Neurotrophic Factor Levels and Improvement of Negative Symptoms in Schizophrenic Patients. *Open Access Maced J Med Sci* (2021) 9:353–357. doi: 10.3889/oamjms.2021.7086
